# Active inference unifies intentional and conflict-resolution imperatives of motor control

**DOI:** 10.1371/journal.pcbi.1010095

**Published:** 2022-06-17

**Authors:** Antonella Maselli, Pablo Lanillos, Giovanni Pezzulo

**Affiliations:** 1 Institute of Cognitive Sciences and Technology, National Research Council (CNR), Rome, Italy; 2 Donders Institute for Brain, Cognition and Behaviour, Artificial Intelligence Department, Radboud University, Nijmegen, The Netherlands; Johns Hopkins University, UNITED STATES

## Abstract

The field of motor control has long focused on the achievement of external goals through action (e.g., reaching and grasping objects). However, recent studies in conditions of multisensory conflict, such as when a subject experiences the rubber hand illusion or embodies an avatar in virtual reality, reveal the presence of unconscious movements that are not goal-directed, but rather aim at resolving multisensory conflicts; for example, by aligning the position of a person’s arm with that of an embodied avatar. This second, conflict-resolution imperative of movement control did not emerge in classical studies of motor adaptation and online corrections, which did not allow movements to reduce the conflicts; and has been largely ignored so far in formal theories. Here, we propose a model of movement control grounded in the theory of active inference that integrates *intentional* and *conflict-resolution* imperatives. We present three simulations showing that the active inference model is able to characterize movements guided by the intention to achieve an external goal, by the necessity to resolve multisensory conflict, or both. Furthermore, our simulations reveal a fundamental difference between the (active) inference underlying *intentional* and *conflict-resolution* imperatives by showing that it is driven by two different (model and sensory) kinds of prediction errors. Finally, our simulations show that when movement is only guided by conflict resolution, the model incorrectly infers that is velocity is zero, as if it was not moving. This result suggests a novel speculative explanation for the fact that people are unaware of their subtle compensatory movements to avoid multisensory conflict. Furthermore, it can potentially help shed light on deficits of motor awareness that arise in psychopathological conditions.

## Author summary

Most of our everyday actions are motivated by the "intentional" imperative to achieve some goal. However, several studies reveal that when we experience a multisensory conflict, such as for example during the rubber hand illusion or when we embody an avatar in virtual reality, we execute movements that are not intentional in the traditional sense, but aim to resolve the conflict (most often, without awareness). Nonetheless, the mechanisms of this second, "conflict resolution" imperative of motor control have received less attention so far. In this article, we advance a model of movement control grounded in the formal theory of active inference that integrates both intentional and conflict-resolution imperatives. Our simulations show that the same computational model can reproduce intentional and conflict-resolution components of action, or both simultaneously. Furthermore, these simulations highlight fundamental differences between movements that are guided by the two imperatives, which map to different error-correction mechanisms of active inference and might contribute to shed light on the reasons why movements guided by conflict-resolution are often executed without awareness.

## 1. Introduction

The field of motor control studies the control of the neuromuscular system for the production of movement, with a specific focus on how to achieve external goals by acting; for example, how we reach and grasp objects. Decades of research have assessed that movement dynamics follow lawful rules that respond to fundamental imperatives (or objective functions), such as the minimization of motor cost and of signal-dependent noise [1–3].

However, there is a further, subtler determinant of movement that has remained largely unaddressed by empirical studies and formal theories. Recent studies show that when participants are exposed to multisensory conflict, such as when they “embody” fake objects (e.g., a rubber hand) and virtual bodies (e.g., avatars), they execute subtle movements that have no obvious goal but are rather aimed at “aligning” with the virtual hands or bodies, hence potentially reducing the conflict and preserving a unitary sense of the self-body that is key to the “embodiment” effects [4–6]. Embodiment (or body ownership) illusions are typically experienced when the view of one’s own body is substituted with the view of a fake body shown in a plausible configuration (i.e., compatible with a first person perspective) and one or multiple streams of congruent multisensory stimuli is at place (e.g. when seeing the fake to be touch and perceiving spatiotemporally aligned somatosensory sensations) [7–10]. Subtle movements have been observed when the embodied fake limb is not aligned with the hidden real body counterpart, both when the embodied limb is static, as in the case of the classic rubber hand illusion (RHI) [11], and during the embodiment of virtual limbs that can be actively controlled, as in immersive virtual reality applications [12]. In the case of the rubber hand illusion, it was observed that when the hidden arm was placed on a horizontally movable board, it tended to move toward the rubber hand, as if in an attempt to reduce the conflict between the real and the rubber hand positions, although participants were not aware of such movements [13]. An analogous behaviour has been observed in participants embodying a virtual static arm not aligned with the physical arm, who tended to exert a steady force in the direction of the virtual arm [14]. Similar effects have been observed when participants have control over the movement of their embodied avatars. For example, in a study, participants were asked to draw lines with their hand while at the same time their embodied avatars were shown to draw ellipses in synch. In this condition, participants tended to engage into elliptical motion patterns without being aware of it [15]. In another study, participants had to perform reaching tasks but they were able to control only one of two joint rotations of their embodied virtual avatars (i.e., the elbow join angle), while the other rotation (i.e., the shoulder join angle) was remotely controlled via software [16]. When the shoulder join angle was manipulated to introduce a spatial mismatch between the real and virtual arms, participants performed movements (shoulder rotations) that reduce the visuo-proprioceptive conflict associated with misalignment, despite these were irrelevant to their reaching task–an effect named the self-avatar follower effect [17].

This growing body of evidence illustrates that the necessity to resolve multisensory conflict and maintain a unitary body representation has subtle effects on motor behaviour that go above and beyond the achievement of external goals. One reason why this topic received limited attention so far is that intentional and conflict-resolution aspects of motor control can only be disentangled in specific experimental settings. While multisensory conflicts about the self-body are common (see below), resolving them through motor action requires a null space in the motor mapping between the agent’s body and its visual counterpart. In other words, it requires that the motor action that compensates for the conflict does not affect the state of the visual representation of the corresponding body part (at least along one degree of freedom). It is only this specific condition that permits compensating multisensory conflicts without interfering with intentional actions—and hence disambiguates the two. This (otherwise rare) condition is usually met during bodily illusions, such as the rubber hand illusion, in which the visual counterpart of the real hand (the rubber hand) stays static no matter what the real hand does. It is also key to the self-avatar follower effect, in which participants can adjust their posture along one degree of freedom without affecting the actions of the avatar that they are controlling. It is important to notice that while gross movements, particularly in the first case, may break the illusion because of the arising visuomotor conflict, subtle motor adjustments could occur with no impact on the illusory state.

Critically, the specific condition that emerges in the above human computer interaction and self-perception studies is not met by classical motor control studies that used multisensory conflicts, such as visuomotor rotations, to study perceptual adaptation and motor learning [18]. Several studies used prisms, mirrors or virtual displays to shift the visual feedback of the arm (or other body parts) during intentional actions, thus introducing multisensory conflict about the self-body [19–22]. For example, during the learning of a new control task, such as controlling a cursor with unusual visuomotor mappings, there can be a spatial mismatch between the real effector (e.g., one’s own hand) and its visual representation (e.g. a colored circle on a screen). However, in this and similar cases, the visuomotor mappings or roto-translations are fixed and cannot be compensated by acting. In other words, any movement of the real hand would result in an analogous movement of its visual representation and hence there is no way to minimize the multisensory conflict by acting. The only way the brain could mitigate the conflict is by sensory adaptation (equivalent to the proprioceptive drift in the rubber hand illusion [11,23]); and, in parallel, by learning a new sensorimotor mapping to keep controlling action accurately.

In sum, the setups adopted in classical motor control studies are useful to probe the learning of novel sensorimotor mappings [24] and the compensatory mechanisms that permit dealing with perturbations [25–27], but do not fully disentangle intentional and conflict-resolution aspects of motor control. Therefore, setups employing the control of an embodied arm via unnatural visuomotor mappings and under perceptual (e.g., visuo-proprioceptive) conflict may be particularly precious in motor control studies, as they provide a unique window to address simultaneously–and disentangle–two imperatives of motor control: an *intentional imperative* that governs goal achievement and a *conflict resolution imperative* that produces automatic motor adjustments not linked to the specific goal but rather functional to coherent self-perception. Importantly, only the first (intentional) imperative figures prominently in mainstream formal theories of motor control such as optimal control theory [2], whereas the latter (conflict resolution) imperative is often ignored in these accounts.

Intriguingly, the framework of active inference proposes an alternative formalization of motor control in biological organisms, which includes natively both intentional and conflict-resolution imperatives [28–37]. In active inference, the *intentional* aspect of motor control is conceptualized as a way to reduce a measure of discrepancy (variational free energy) between desired states (e.g., hand on target) and sensed states (e.g., hand far from target). In parallel, however, the movements of an active inference agent can also be guided by the imperative to *resolve any multisensory conflict* that arises during perception; for example, if a person has incongruent visual and proprioceptive information about the position of one’s hand.

Strong multisensory conflicts (e.g., between visual and proprioceptive streams) are uncommon in daily life conditions, but common during body ownership illusions. Theoretical accounts hold that bodily illusions arise because people infer that there is a unique cause or source for all their bodily sensations, including visual stimuli from a fake hand or body [9,38–40]; see [8,41] for a discussion of this hypothesis. For example, in the rubber hand illusion [11], people might infer that visual and proprioceptive sensations should be congruent, because they have a common cause (i.e., they come from the same hand)–whereas in reality, visual information comes from the fake hand, which is misplaced with respect to the real hand, therefore creating a visuo-proprioceptive conflict. While for illustrative purposes we emphasized the rubber hand illusion, similar multisensory conflicts could arise across several other bodily illusions that we have reviewed above.

Importantly, an active inference agent can resolve the multisensory conflict that can arise in the rubber hand and other body ownership illusions in two ways: through perceptual recalibration and compensatory movements, respectively. The first way corresponds to changing the internal estimate of the hand position to lie in between the real and the fake hands, giving rise to the "proprioceptive drift" commonly observed during the rubber hand illusion and more broadly, to perceptual recalibration observed in visuomotor conflict studies [42]. Most Bayesian accounts of bodily illusions [23,43,44] focus on these perceptual phenomena, in analogy to (Bayesian) explanations of multisensory perception and cue integration [45]. However, the framework of active inference is not just concerned with perception but also with action dynamics and it suggests another way to resolve multisensory conflict. This second way consists in making a compensatory movement that cancels out the conflict. In other words, an active inference agent can change the hand position to get closer to the fake hand–which, as noticed above, is exactly what happens if a person’s hand is allowed to move during the rubber hand illusion [13,14,17]. Both the mechanisms that we have illustrated–one based on perceptual recalibration and the other based on compensatory movements–can be accaunted by a common mathematical operation in active inference: the minimization of variational free energy (VFE), see the Methods section [29]. Furthermore, the two are strictly linked to one another. The proprioceptive drift occurs whenever there is a misalignment of the real and the embodied limb and the agent updates its (multisensory) belief about the position of the arm. If no movement is allowed, as in the rubber hand illusion, this results in a perceptual recalibration of the perceived arm location at an intermediate location, which is usually biased towards the fake arm given the visual dominance. When the movement is allowed, the proprioceptive drift is still present but it also triggers a compensatory action that brings the real hands towards the (inferred) position of the arm–i.e., towards the fake body–and can eventually cancel out the sensory conflict (and even the proprioceptive drift).

In sum, this example clarifies that, in active inference, a movement can arise not just to achieve an external goal (intentional imperative), but also to resolve a multisensory conflict, in the absence of an external goal (conflict resolution imperative). While the two imperatives can be in play simultaneously, they can be distinguished conceptually and mechanistically. Indeed, both intentional and conflict resolution imperatives resolve a discrepancy, but this discrepancy is different in the two cases. When an agent has an external goal, the desired goal state drives perceptual inference away from sensory estimates, giving rise to sensory prediction errors. The intentional imperative amounts to triggering an action to resolve these prediction errors. However, sensory prediction errors can arise even in the absence of an external goal; for example, when an agent has incongruent visual and proprioceptive information about the position of the hand. The conflict resolution imperative amounts to triggering an action to resolve this latter type of prediction error.

The active inference framework is potentially able to explain–and disentangle–intentional and conflict resolution imperatives of movement control. However, active inference models of movement control have been mostly used to describe goal directed actions [46–48] and have rarely investigated the more subtle and elusive aspects of motor behaviour that are due to conflict resolution (but see [14,31]). The main contribution of this paper is addressing the two (intentional and conflict resolution) imperatives of movement control simultaneously in the same active inference model. This permits shedding light on the fundamental differences in motor control and awareness between movements that achieve the two imperatives–or the fact that people are generally aware of the intentional movements they execute but are often unaware of their automatic motor adjustments that they execute to resolve conflicts.

In this paper, we present an active inference model of arm perception and control that integrates two imperatives: an *intentional imperative* that governs the achievement of goals and a *conflict-resolution imperative* that permits avoiding multisensory inconsistencies, such as those that derive during bodily illusions. We exemplify the functioning of the active inference model by simulating three tasks that highlight the different imperatives of movement control: intentional (first simulation), conflict-resolution (second simulation) or both (third simulation). In the first simulation, the agent is assigned an external goal: it has to perform a reaching action under visuo-proprioceptive guidance. In the second simulation, corresponding to a rubber hand illusion, the agent has no external goal but has to infer its own body state (here, arm location) when exposed to a multisensory (here, visuo-proprioceptive) conflict about the hand position. In the third simulation, analogous to studies of adaptation to novel sensorimotor mappings, the agent has to perform a goal-directed reaching task (as in the first task) while it experiences a multisensory conflict (as in the second task).

This study makes three contributions. First, it shows that the same active inference model can simultaneously address different aspects of motor behaviour that are usually addressed independently from one another. Second, it shows that despite their similarities at the behavioural levels, the movements that achieve intentional and conflict-resolution imperatives are driven by two different (model and sensory) kinds of prediction errors. Third, it shows that it is only in the case of intentional actions that the active inference agent correctly infers its velocity. Rather, if movement is only guided by conflict resolution, the agent may incorrectly infer that its velocity is zero, as if it was not moving. This latter finding provides a novel explanation for the fact that while people are aware of their intentional movements, they are generally unaware of the motor adjustments that they execute to resolve multisensory conflicts.

## 2. Methods

In this section, we describe the simulation setup and the active inference agent that we use in the three simulations of reaching movements with no multisensory conflict (first simulation), multisensory conflict with no movement (second simulation) and reaching movement with multisensory conflict (third simulation).

### 2.1. Simulation setup

**Fig 1:**
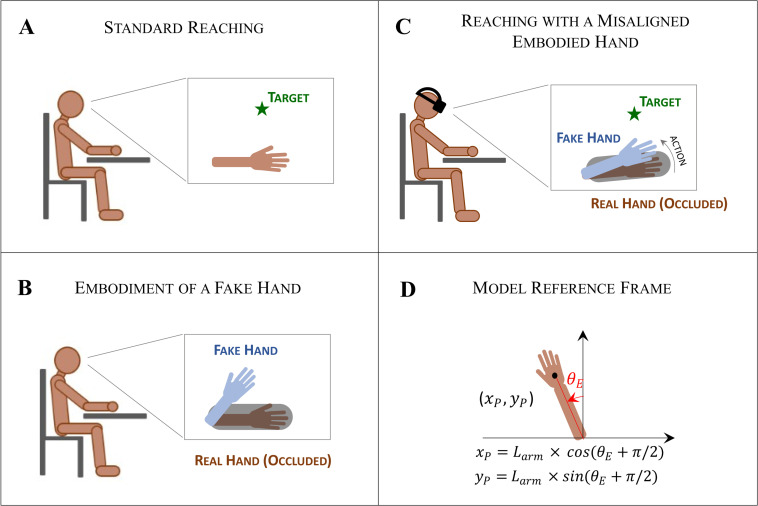
The simulation setup. (A-C) The three simulations included in the study. In all cases, the agent is seated with the forearm on the table. (A) In the first simulation, the agent has direct visual access to the real hand or equivalently, to a virtual hand projected to spatially overlap the actual hand position. (B) In the second simulation, the agent embodies a static fake hand placed in a configuration different from the real hand, hidden from view. (C) In the third simulation, the agent embodies a virtual arm seen through a stereoscopic visor and has to reach a target; the simulation includes a manipulation of the visuomotor mapping that, as the agent moves, introduces a spatial misalignment between the two hands. (D) In all the simulations, the agent is in control of one degree of freedom of the elbow, which can rotate along the vertical axis. The agent state is then uniquely described by the elbow joint angle *θ_E_*, with the corresponding visual representation of the end-effector specified in Cartesian coordinates (*x_p_, y_p_*).

The setup that we use for the three simulations is illustrated in Fig 1A–1C. In all cases the agent is seated with the arm on a table and can move the arm by rotating the elbow around the vertical axis. In this one-dimensional control problem, the agent’s configuration is fully described by the elbow’s joint angle, *θ_E_* (Fig 1D). In the first simulation, the visual input comes from the direct vision of the real hand (Fig 1A) and the agent is instructed to reach a target. In the second simulation, the agent has direct view of a rubber hand placed in different configuration with respect to real hand, which is conceal from view (Fig 1B), with any motor task assigned. In the third simulations, the agent wears a stereoscopic visor displaying a virtual arm and has to reach an assigned target; the virtual hand can be controlled, but with an unnatural visuomotor mapping that introduces a misalignment between the two hands (Fig 1C). In the second and third simulations, the fake arm contributes to the system dynamics as a visual sensory input that replaces the visual input from the hidden real hand, hence creating a multisensory conflict when the two hands are not aligned. In the model, the visual encoding of the hand location is represented in Cartesian coordinates, as an approximation to the information that is processed by the retina. In the assumption that the visual input arises from the real hand, the Cartesian coordinates depend uniquely on the elbow joint angle, as in Fig 1D.

### 2.2. Active inference agent

We realized an active inference agent that is able to reproduce at a qualitative level the behaviour commonly observed in the lab under conditions equivalent to the three aforementioned tasks. Below, we describe the two main elements that are necessary to set up an active inference simulation. The former is the so-called "generative process" of active inference, which corresponds to the agent’s environment, or the system with which it interacts by receiving inputs and sending control actions. Here, it corresponds to the agent’s arm and its dynamics. The latter is the so-called "generative model", which corresponds to the agent’s model of the system. The implementation of the perception-action loop adopted for simulating the dynamics of the system (both the environment and the internal representation that the agent has of it) is described next. Finally, we provide a formal introduction to active inference in S1 Appendix. The interested reader is also referred to other publications that provide a comprehensive treatment of active inference for motor control [14,29,30,37,48,49].

### 2.3. Generative process

**Fig 2:**
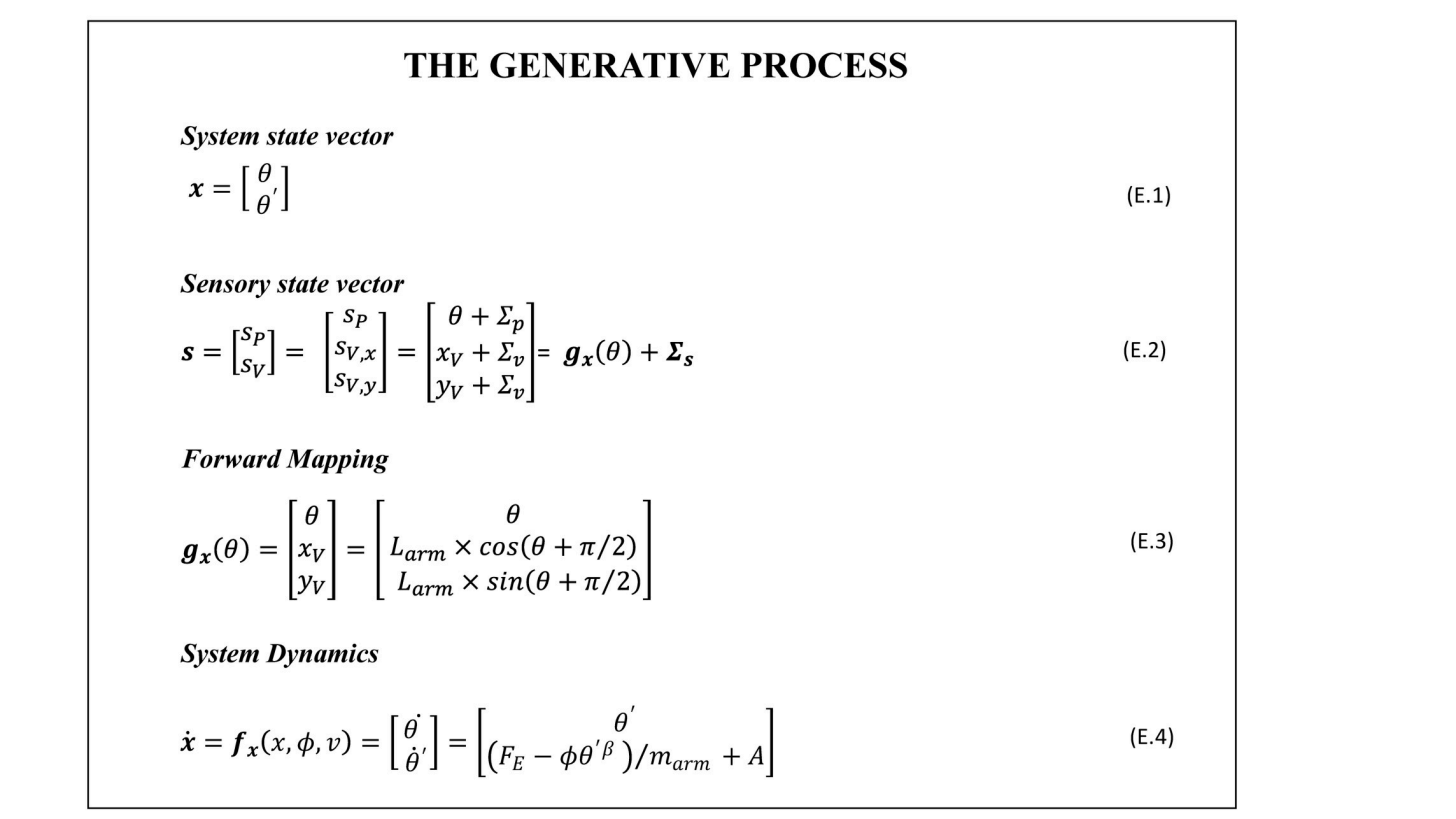
Mathematical description of the generative process. The system state vector (Eq E.1) describes the system in generalized coordinates at the first order. The sensory state vector (Eq E.2) maps the system states into the sensory input (here proprioception and vision). The forward mapping (Eq E.3) describes how the system state vector maps into sensory input. The system dynamics (Eq E.4) is expressed as a set of differential equations that describe the expected temporal unfolding of the system state. The arm dynamics is approximated as a damped system driven by a combination of external forces (F_E_) and agent’s actions (A); in Eq E.4 *m_arm_* represents the forearm mass and *ϕ* the viscosity constant of the damping. We assume a power dependence between damping and velocity, with *β = 0.5*, which allows reaching plausible velocity profiles.

The generative process is a mathematical description of the system with which the agent interacts by receiving inputs and sending control actions; namely, the agent’s arm and its dynamics. The generative process includes four elements: (i) a *system state vector* that describes the real configuration of the system at any given time; (ii) a *sensory state vector* that describes the sensory input that informs the agent about the current state of the system; (iii) the *forward mapping* that maps the system’s state into the sensory input available to the agent and (iv) the *system dynamics*. Below we introduce these four elements in order; see also Fig 2 for a detailed mathematical description.

The ***system state vector*** (Eq E.1 in Fig 2) is described in generalized coordinates, akin to previous implementations of active inference [28–30]. In essence, generalized coordinates are adopted here to describe the dynamical state of the system at a given point in time, therefore including the time derivatives of the state configuration at different orders. Note that such description entails by definition predictions about how the system will evolve in the near future. In our model, we approximate the state description at the first order, so as the vector specifying at a given time the location of the arm, *θ*, and its velocity, *θ*′.

The ***sensory state vector*** (Eq E.2 in Fig 2) is defined by a forward model describing the system state as registered by the different sensory channels. In our model the agent gathers information about the system’s state (in this case about its own posture) through proprioception and vision. The hand configuration sensed through proprioception can be treated as an actual measure of the elbow angle, *θ*, as sampled by proprioceptive receptors (e.g. neuromuscular spindles and Golgi tendon organs), with an associated sensory noise Σ_*P*_. The visual input provides information about the arm configuration in retinal coordinates. For simplicity, we take as visual input the location of the palm centre in the 2D Cartesian reference frame illustrated in Fig 1D. In the second and third simulations, we assume that the vision of the virtual hand is processed as a visual cue originating from the real hand (which the agent cannot see), an assumption that holds only if a sustained ownership illusion is taking place. The visual input can be therefore treated as a measure of the virtual hand location (*x*_*V*_, *y*_*V*_) sampled with an associated sensory noise Σ_*V*_. For simplicity, we assume the visual noise to be the same along both directions of the Cartesian system.

The ***forward mapping*** (***g***_***x***_) (Eq E.3 in Fig 2) maps the system state vector into the corresponding description of the system state in the reference frames of the sensory channels available (here, proprioception and vision). As mentioned above, we assume that proprioceptive receptors directly measure the joint angle, so that the corresponding forward mapping is the identity function. Rather, vision encodes the hand position in Cartesian coordinates, which can be derived from the elbow joint angle by considering the geometry of the system, shown in Fig 1D. This forward mapping can be then used to compute the sensory state vector corresponding to a given system state, as in Eq E.2.

The ***system dynamics*** (Eq E.4 in Fig 2) describes how the system evolves under the effect of external forces and internally generated actions, in accordance with the underlying physical laws. It can be formulated as a set of differential equations describing how the system state vector varies in time. It is generally expressed in the form: $\dot{x}=f(x,\alpha ,v)$, where **α** denotes a set of model parameters (characteristic of the system physical structure) and ***v*** a set of drivers or attractors of the system dynamics. These attractors could be present either in the form of external forces acting on the system, ***F***_***E***_, or in the form of actions internally generated and executed by the agent, ***A***. In our model, the system dynamics describes the temporal evolution of the real arm configuration. We approximate the arm dynamics as a damped system, controlled in velocity (i.e., the equations provide an expression for changes in velocity) exclusively through internally generated actions–hence we assume contributions from external forces to be null. Note that the action ***A*** is expressed as the variation of the joint angle velocity in unit time, therefore it has the dimension of a joint angle acceleration.

### 2.4. Generative model

**Fig 3:**
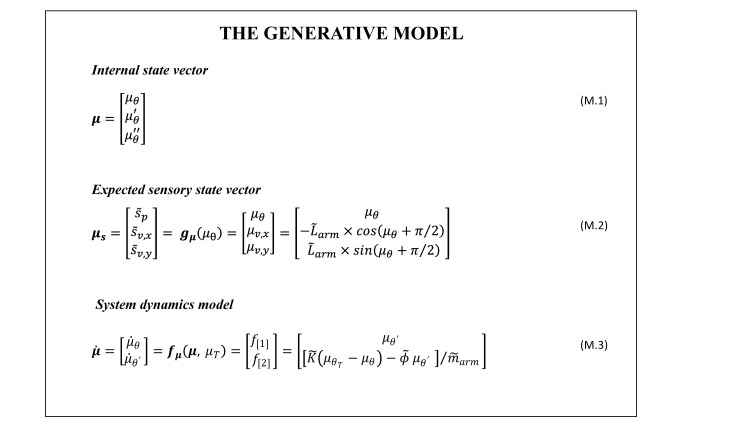
Mathematical description of generative model, i.e. the internal model the agent holds of the system and its dynamics, for the 1D model shown in Fig 1. The agent represents its own state via the internal state vector that describes the system in generalized coordinates at the second order (Eq M.1). The forward model, ***g**_**μ**_(μ_θ_)*, describes how the agent forms an estimate of the expected sensory input based on the inferred system state, ***μ**_**s**_* (Eq M.2). The model of the system dynamics allows the agent to predict the temporal evolution of its own state. In particular, the agent in our model entails a representation of reaching actions as instantiations of a desired state, $\mu_{\theta_{T}}$, which acts as an attractor. The dynamics is then assumed to follow that of a damped oscillator (Eq M.3), with *K* representing the elastic constant controlling the attraction strength, *ϕ* the viscosity constant of the damping, and *m_arm_* the mass of the agent forearm. Note that the model in Eq M.3 describes also states in which the agent does not intend to move; in this case the desired state is to be set to the current state, i.e. $\mu_{\theta_{T}}=\mu_{\theta }$.

The generative model is the agent’s internal representation of the environment and of its dynamics. It includes three components that share resemblances with (but are not identical to) the corresponding elements of the generative process. These include (i) an estimate of the agent’s current state in the form of an *internal state vector*, i.e., the belief, (ii) an estimate of the expected sensory feedback associated with the inferred current state, i.e. the *expected sensory vector*, and (iii) a *system dynamic model* that describes the agent’s representation of how the system state evolve in time given a specific inferred configuration. Below we introduce these three elements in order; see also Fig 3 for a detailed mathematical description.

The ***internal state vector*** (Eq M.1 in Fig 3) is an estimate of the agent’s current state and it is defined by the position, velocity and acceleration of the inferred configuration. The reason to use up to the second order derivative is that the differential equations that compute the dynamics of the system return the first and the second order derivatives of the state. Thus, we need to track the second order state estimate (acceleration) to be able to compute the prediction error. For simplicity, and in keeping with previous active inference implementations [28–30], we assume that the agent models the system state in the same domain as the system state vector in the generative process. This assumption allows mirroring the expressions of the generative processes in the generative model.

We assume that the agent’s representation of the system’s structure and dynamics resembles the generative process counterparts. Specifically, we assume that the agent computes an ***expected sensory vector*** (***μ***_***s***_) (Eq M.2 in Fig 3) with an internal representation of the forward mapping between the system state and the corresponding sensory input, given by ***g***_**μ**_(*μ*_*θ*_). Note that ***μ***_***s***_ is to be compared with the afferent sensory feedback ***s*** (in Eq E.2 in Fig 2) for computing sensory prediction errors, and shall be therefore defined in the same domain as ***s***.

Furthermore, we assume that the agent’s generative model includes an internal representation of the **system dynamics** (Eq M.3 in Fig 3). Given that we are interested in simulating reaching actions in the absence of external forces, we follow previous active inference implementations that treat the intended target as a point attractor. This is done by including in the internal model a force driving the hand towards the target, i.e. a force directed along the vector from the current hand location to the target, and proportional to the hand-target distance (Eq M.3 in Fig 3). The internal model includes also a damping factor ($-\tilde{\phi }\;\mu_{\theta^{\prime }}$, which prevents the velocity to increase indefinitely), necessary to reproduce plausible reaching dynamics in which the hand does not overshot the target and oscillate around it. Please note that the presence of an intended goal, or desired state, in the agent’s generative model marks an important difference with the generative process in which the attractor is not present–and permits the agent to autonomously pursue its intended goals rather than passively following environmental dynamics.

Performing a (visually-guided) reaching movement requires the to-be-reached target to be associated with a corresponding system configuration, *x*_*T*_, for which the agent’s hand is on the object. In the current case, this will be a specific elbow angle: *x*_*T*_ = θ_*T*_. The agent, however, does not have direct access to the real environment variables, and must therefore rely on the estimates of the target location, $\mu_{V}=[\mu_{x_{T}},\mu_{y_{T}}]$, and of the corresponding system configuration, $\mu_{\theta_{T}}$. To infer the latter, the agent can adopt the inverse of the forward model plausibly learned based on the lifelong multisensory experience of the body. In our implementation, for simplicity, we skip this step and set the target directly as a desired system configuration, $\mu_{\theta_{T}}$.

Finally, the system dynamics model (Eq M.3 in Fig 3) permits also characterizing a “resting” condition in which the agent does not intend to move. In this setting, being at rest could be modeled assuming that the agent’s goal is to remain in the current state–which corresponds to setting the attractor to the current state estimate, i.e. $\mu_{\theta_{T}}=\mu_{\theta }$. When the active inference agent is in the “resting” condition, the internal model of the system dynamics does not trigger any movement. However, how we will discuss below, the agent can still generate movements unintentionally, to resolve multisensory conflict (see the second simulation below).

### 2.5. Action-perception loop of the active inference agent

**Fig 4:**
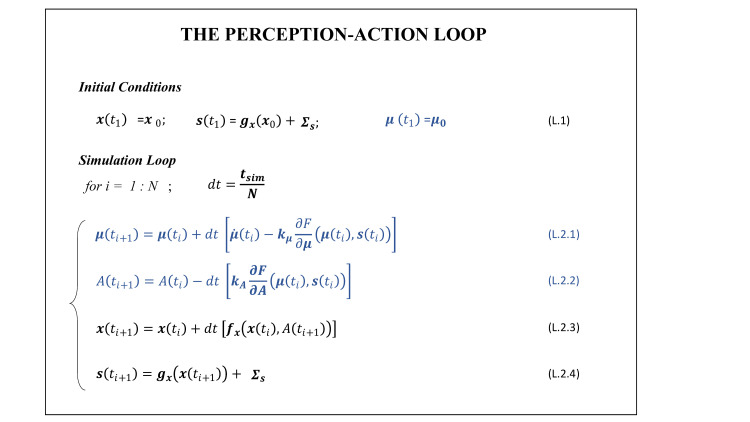
Perception-action loop of the active inference agent. The variables and equations in black (blue) are from the generative process (generative model), thus represent the real world (internal representations the agent holds about the system state and its dynamics). See the main text for a detailed explanation.

In active inference, both perception and action arise from a common mathematical operation: the minimization of variational free energy (VFE) [29]. In this setting, the agent’s perception and action dynamics can be simulated by computing the evolution of the coupled dynamical system in Fig 4, Eq L2.1-L.2.4. Once the initial conditions (i.e., the system and internal state vectors and the corresponding sensory state vectors) are set (Eq L.1 in Fig 4), the evolution in time of the system can be computed by iterating on discretized time intervals, with the desired resolution set by selecting the number of iterations, *N*, in relation to the simulation duration, *t*_*sim*_. At each time step, the inferred state of the hand and the action selected by the agent are calculated using Eq L.2.1 and L.2.2, respectively. Note that the inferred state evolution depends both on the expected dynamics and on the minimization of the VFE via gradient descent, while the action’s update is only driven by the gradient descent of free energy. As we discuss in detail below, although the action *A* is a scalar, it results from the contribution of two components, which minimize VFE by minimizing the proprioceptive and the visual prediction errors, respectively. Next, the effect of the action on the environment is calculated by updating the system state vector, using the real dynamics (Eq L.2.3). Finally, the sensory input vector associated to the system state vector is obtained using Eq L.2.4; this is the input that will be used in the next iteration by the agent to infer the system state and select an action.

Importantly, the simulation has to take into account the fact that the intrinsic dynamics of the system and the free energy minimization could in principle unfold at different time scales. If the intrinsic dynamics of the system were much faster, then it would not be influenced by the free energy minimization; and vice versa if the free energy minimization were too fast, it could create motor instabilities. To ensure that the intrinsic dynamics of the system and the free energy minimization unfold on compatible timescales, it is necessary to introduce some additional parameters–the perceptual gains, ***k***_***μ***_ = [*k*_*μ*_, *k*_*μ*′_, *k*_*μ*′′_] and the action gain *k*_*A*_–that modulate the rate of gradient descent of free energy. Please note that later we will split the action gain *k*_*A*_ into two actions gains, which modulate the minimization of the proprioceptive prediction error (*k_A,p_*) and of the visual prediction error (*k*_*A*,*v*_), respectively.

To solve the coupled evolution of perceptual inference and motor control, we therefore need to compute the VFE of the system F, and next the gradients of F both with respect to the components of the internal state estimate ***μ*** (here *μ*_*θ*_ and *μ*_*θ*′_), and to the action *A*.

**Fig 5:**
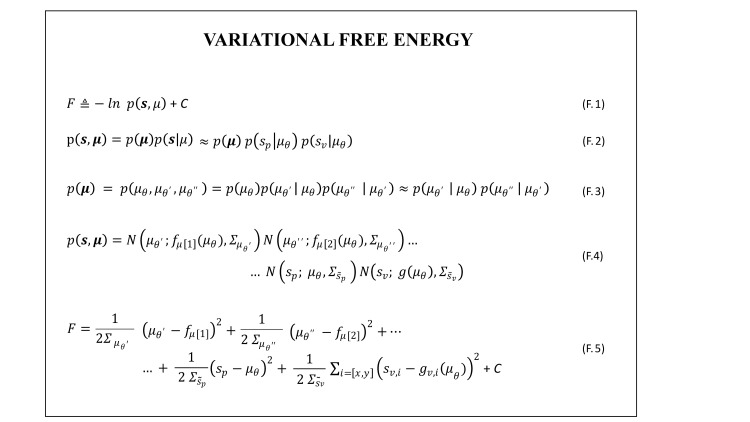
Derivation, under the Laplace approximation, of the variational free energy (VFE), F, for the active inference agent. See S1 Appendix for the detailed derivation of Eq F.1, in which F is expressed in terms of the Laplace encoded energy, and C is a term assumed here to be a constant that encodes the optimal variance (omitted for clarity as it will not be used for computing the gradients as it does not depend on the internal state ***μ*** nor the action *A*). *p(**s**_**p**_|**μ***) and *p(**s**_**v**_|**μ***) are the proprioception and visual likelihood given the internal belief *μ_θ_*. $p({\mu_{\theta },\mu }_{\theta }^{\prime }\mu_{\theta }^{\prime \prime })$ is the joint probability of the internal state vector up to 2^nd^ order, which can be expressed in terms of conditional probabilities on the system state as expected from the internal dynamical model. Both the sensory state likelihood and the conditional probabilities are approximated as Gaussians centred on the expected sensory state and the expected value of the dynamics at the different orders (from Eq M.3 in Fig 3) respectively. Please see the main text for a more detailed explanation.

As discussed in previous studies [29,30,37,49], under the Laplace approximation, the gradient of F with respect to the internal state is equivalent to the gradient of the Laplace encoded energy, −log *p*(***s*,*μ***) (Eq F.1 in Fig 5, whose derivation is summarized in S1 Appendix). For the problem under scrutiny, we can work out a derivable expression of −log *p*(***s*,*μ***). As a first step, we work out an expression for *p*(***s*,*μ***), assuming that (i) the likelihood for receiving a sensory input in a given modality is a normal distribution centred on the corresponding state estimate (here *μ*_*θ*_ for proprioception, and ***g***(*μ*_*θ*_) for vision, with corresponding expected sensory noises $\Sigma_{{\tilde{s}}_{p}}$ and $\Sigma_{{\tilde{s}}_{v}}),$ and (ii) that the two sensory input are independent (leading to Eq F.2-F.4 in Fig 5).

Crucially, the prior belief about the current state of the system, *p*(***μ***), is informed by the expected system dynamics. In particular, given the adopted representation of the internal state (Eq M.1 in Fig 3), *p*(***μ***) = *p*(*μ*_*θ*_, *μ*_*θ*′_, *μ*_*θ*′′_) could be worked out as the product of conditional probabilities, and written as *p*(***μ***) = *p*(*μ*_*θ*′_|*μ*_*θ*_) *p*(*μ*_*θ*′′_|*μ*_*θ*′_), as in Eq F.3 in Fig 5. This holds under the assumption that, by default, any initial state system is considered equally probable (i.e., *p*(*μ*_*θ*_) is uniform). In order to work out an expression for *p*(***μ***), we shall consider that the expected value of each dynamical order of the internal state vector is informed by the corresponding time derivative of the state at the order below (as from Eq M.3 in Fig 3): thus, 〈*μ*_*θ*′_〉 and 〈*μ*_*θ*′′_〉 (where the 〈.〉 indicates the expected value) are informed by $\dot{\mu_{\theta }}=f_{\left[1\right]}$ and ${\dot{\mu_{\theta^{\prime }}}=f}_{\left[2\right]}$, respectively (see Eq M.3 in Fig 3). Considering prior predictions as noisy processes, we can adopt Gaussian representations centered on the corresponding expected values, with the standard deviation representing the agent’s uncertainty on the internal model, $\Sigma_{\mu_{\theta^{\prime }}}$ and $\Sigma_{\mu_{\theta^{\prime \prime }}}$. Plugging all the normal distributions in Eq F.2 (Eq F.4 in Fig 5) and applying the basic rules of logarithms we obtain an analytical expression for F (Eq F.5 in Fig 5). The latter shall be derived with respect to the perceptual estimates and to the action and plugged in the perception-action loop computation.

**Fig 6:**
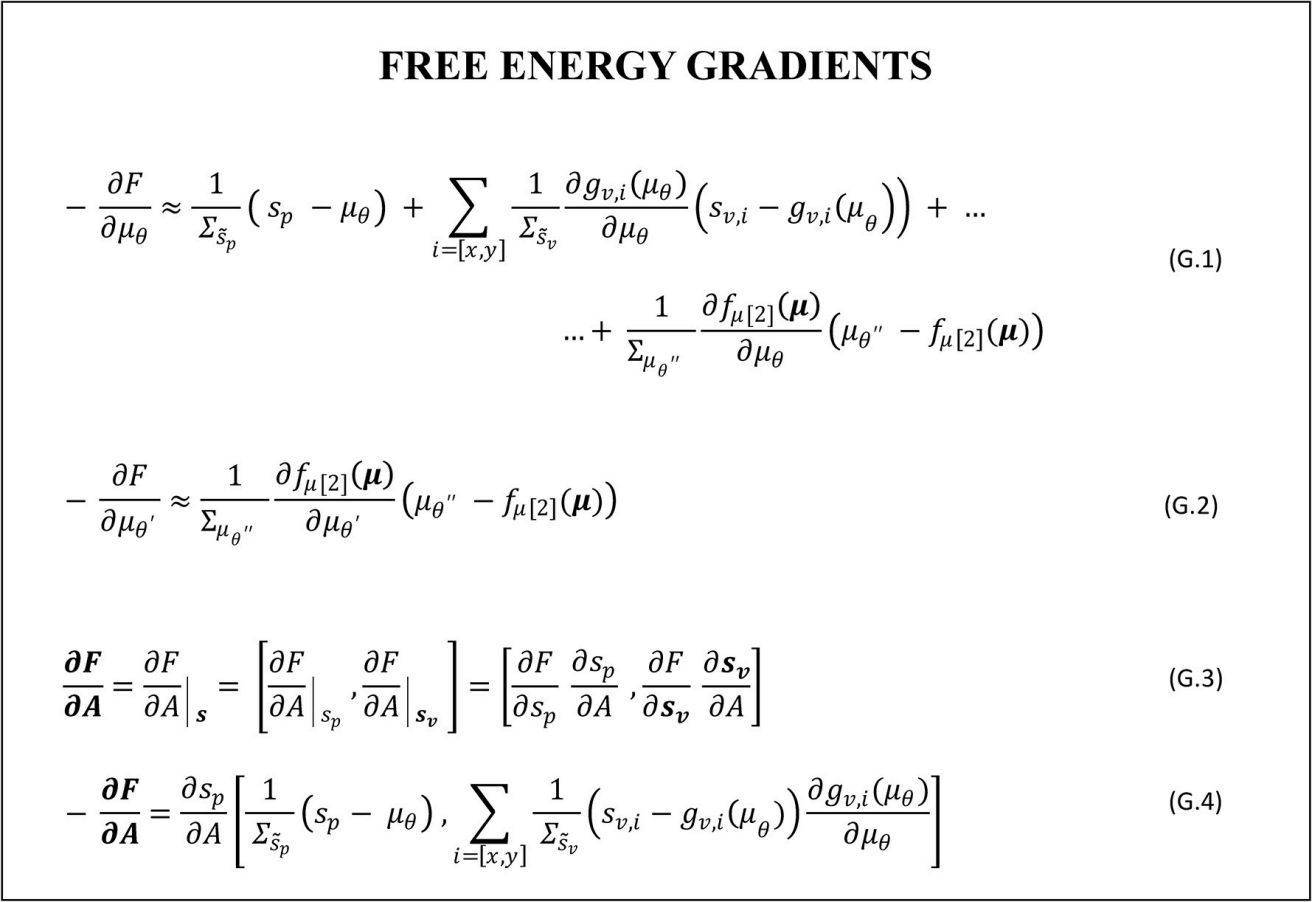
Derivation of the free energy gradients for the active inference agent. Please see the main text for a detailed explanation.

In the specific formulation adopted for our problem the variational free energy takes the simple form given in Eq F.5 in Fig 5. The contribution of the free energy gradient descents contributing to the estimate of the elbow joint angle and of its velocity (via Eq L.2.1 in Fig 4) can be easily derived and are given in Eq G.1 and Eq G.2 in Fig 6, respectively. When it comes to derive F with respect to *A*, however, one need to face the issue that actions are not explicitly represented in the internal model and therefore *A* does not enter in the formulation of F. To overcome this apparent deadlock, active inference assumes that agents hold an implicit knowledge of how actions map into changes in sensory states, formally of ∂**s**/∂A, which is assumed to be hardwired or learned throughout lifespan [30]. This allows deriving the gradient of F with respect to *A* by minimizing the free energy with respect to the sensory state vector, as in the first equivalence in Eq G.3 in Fig 6. Note that doing so, the gradient of F with respect to *A* results a two dimensional vector, with the two components associated with the minimization through action of the proprioceptive and visual prediction errors. In fact, given that we are interested in simulating visually guided actions, we assume that the agent selects actions to minimize both visual and proprioceptive errors (second equivalence in Eq G.3 in Fig 6), as previously done in [48]. Accordingly, the gains for the free energy minimization through action, should include two components: ***k***_***A***_ = [*k*_*A*,*p*_, *k*_*A*,*v*_]. Note that this differs from most active inference models of motor control, which assume that the agent selects actions to minimize proprioceptive errors only.

A fundamental assumption of the active inference formulation is that the agent holds an intrinsic knowledge of ∂*s*_*p*_/∂A, implemented by reflex arcs, and more specifically by a chain of reflexes allowing to automatically (with no motor planning) achieve a given change in the agent’s state as sensed by proprioception [30]. Still, in actual implementations of active inference, it is necessary to derive a mathematical representation for this term. Considering that the relation is intrinsically rooted in the agent’s knowledge of its body structure and actuators dynamics, it is possible to derive a plausible relation based on the internal representation of the system dynamics. Advanced learning techniques with function approximators are out of the scope of this work [50]. For the specific implementation adopted here, *A* is defined as a change in angular velocity, as from Eq E.4 in Fig 2. We can then rewrite the dynamics of the velocity given in Eq M.3 in Fig 3, by setting ${\dot{\mu }}_{\theta^{\prime }}$ as an action *A*, and by expressing *μ*_*θ*_ as a function of *A*. Considering that the proprioceptive input *s*_*p*_ is here assumed to provide a noisy measure of the elbow joint angle *θ*, internally represented as *μ*_*θ*_, one plausible expression for *∂*s*_*p*_/∂A* can be derived computing ∂*μ*_*θ*_/∂A from the expression derived as above, which return ${-\tilde{m}}_{arm}/\tilde{K}$ as a result. Given this, we obtained a computable expression for ∂F/∂A as in Eq G.4 in Fig 6.

The expressions derived for the VFE gradients (Eq G.1-G.4 in Fig 6) clearly show how both perceptual inference and actions “move” in the direction that suppresses prediction errors. However, while action is driven exclusively by the need to suppress the sensory prediction errors (***ε***_*s*_ = ***s***−*g*(***μ***)), perceptual inference is driven both by sensory prediction errors ***ε***_*s*_ and by model prediction errors $\varepsilon_{m}={\mu^{\prime }}_{\left[2:3\right]}-f_{x}(\mu )$. Note that in our model the first component of the model error is null by definition (Eq M.3 in Fig 3), thus its contribution does not appear in Eq G2 in Fig 6. This is a crucial point because, as it will be shown in the following, these two predictions errors–which enter the derivation of action through *μ*_*θ*_–underlie two different imperatives of motor behavior, which might have different demands in terms of motor awareness (see the Discussion for a more detailed discussion on motor awareness).

It is worth to clarify the reason why the internal state vector should include the second order *μ_θ′′_*, which is related to the computation of model dynamics errors. Model dynamics errors represent the offsets between the estimated changes in the predicted system (as informed by sensory input) and the corresponding changes predicted by the internal dynamics. This implies, in the general case, that the internal estimate of the system state should extend to one order higher with respect to the one at which the system dynamics is internally modeled. Thus, because in our case the dynamics is formulated at the first order, i.e. as changes in velocity (Eq M.3, Fig 3), we need to include an estimate for acceleration in the internal state vector (Eq M.1, Fig 3). In the model implementation we computed the *μ*_*θ*′′_ temporal evolution by setting changes in the expected acceleration to random fluctuations, a standard choice adopted in previous active inference models; see [34].

### 2.6. Generative process and model used in the three simulations

The characterization of the generative process was fixed and kept the same for all the three simulations. The mass and length of the real arm were set to the average corresponding values of adult human forearms: *L*_*arm*_ = 45 *cm* and *m*_*arm*_ = 1.5 *kg*. The noise levels associated the sensory input in the generative process are set to plausible values, according to the known accuracy for proprioception and vision: we set Σ_*p*_ = 0.1 rad (corresponding to 5°) as the noise associated with the joint angle decoding from proprioception, and Σ_*v*_ = 1 cm for the visual encoding of the hand’s centre position. For all three cases, we follow the simulations for a duration of *t*_*sim*_ = 20 seconds, with a temporal resolution, *dt*, of 10 milliseconds. The parameters of the system dynamics (Eq E.4 in Fig 2) were set to *ϕ* = 4 and β = 0.5. Differently from previous implementations of reaching actions, we considered a non-linear dependence of the damping on the arm velocity, to avoid having critical damping (stopping at the target without oscillating around it) achieved over long time scales.

We designed a unique generative model and used the same set of parameters for the three simulations. The only exception to this are the internal estimates for the noise of the sensory inputs ($\Sigma_{{\tilde{s}}_{v}}$ and $\Sigma_{{\tilde{s}}_{p}}$) and the action gains (*k*_*A*,*p*_ and *k*_*A*,*v*_), to account for the different demands of the simulations that include (first and third) or not include (second) an explicit motor task. In the design of the generative model, we assumed that the agent has a non-biased representation of its body (i.e., ${\tilde{L}}_{arm}=L_{arm}$ and ${\tilde{m}}_{arm}=m_{arm}$). Next, we fixed the parameters adopted for the internal model of the system dynamics were set $\tilde{\phi }$ = 4 and $\tilde{K}=1.3$ for all simulations, reflecting the assumption that the agent has a robust model of its dynamics. Note that, while the damping parameter $\tilde{\phi }$ could faithfully represent its counterpart in the generative process, the elastic parameter does not have such a counterpart. Said that, it is important to notice that $\tilde{K}$ governs the strength of the attractor (in the damped oscillator the elastic force), thus paralleling the role that action A plays in the dynamics of the generative model. For this reason, it is to be kept in mind that there is a direct interplay between the values of $\tilde{K}$ and of the action gain, ***k***_***A***_, which in essence governs how fast the model descends the free energy gradient.

As mentioned above, the relative values of these gains need to be tuned with respect to the intrinsic dynamics of the system and according to the temporal resolution of the simulation. Their specific values then reflect the need to calibrate the relative rates at which energy minimization for perception and action occurs on the top of the system dynamics. Here we assume that *k*_*μ*_ = 0.1, *k*_*μ*′_ = 0.01 and *k*_*μ*′′_ = 0.001. Similarly, we fix the uncertainties associated with the internal model, $\Sigma_{\mu_{\theta^{\prime }}}$ and $\Sigma_{\mu_{\theta^{\prime \prime }}}$, to 0.01 and 0.1 respectively. We provide the specific values for sensory noises ($\Sigma_{{\tilde{s}}_{v}}$ and $\Sigma_{{\tilde{s}}_{p}}$) and action gains (*k*_*A*,*p*_ and *k*_*A*,*v*_) in the description of the simulations.

## 3. Results

In this section, we use the proposed active inference agent and the simulation setup described in the Methods section to simulate three tasks that highlight the different imperatives of movement control: intentional (first simulation), conflict-resolution (second simulation) or both (third simulation). Our simulations show that active inference unifies in a common framework both the control of intentional actions and the execution of movements that resolve multisensory conflicts in the absence of external goals. Furthermore, our simulations highlight that acting intentionally and acting to resolve conflicts imply different dynamics of the hidden states of the agent’s generative model. In turn, these differences might be relevant to understand why only the former kind of (intentional) action is generally associated to awareness.

### 3.1. First simulation: reaching a target in space

In the first simulation, the active inference agent has to reach a fixed target location with its right hand. In this simulation, there is no multisensory conflict: visual and proprioceptive inputs are consistent with one another. We set the starting elbow joint angle of the agent at *θ* = 0, which corresponds to having the forearm parallel to the midsagittal plane. Furthermore, we set the target to the location that the hand would occupy when the elbow joint angle is θ_*T*_ = *π*/3, thus at [*x*_*T*_, *y*_*T*_], derived as in Fig 1E. Please note that setting the target location a function of a plausible elbow joint angle ensures that the task is achievable in the 1DoF problem considered here.

The internal estimates for the sensory noise are assumed to be unbiased with respect to the sensory noises characterizing the generative process, thus $\Sigma_{{\tilde{s}}_{p}}=0.1$ and $\Sigma_{{\tilde{s}}_{v}}=0.01$. We further assume that the action gains driven by proprioceptive and visual predictions errors are the same, thus *k*_*A*,*p*_ = *k*_*A*,*v*_ = 0.3. Finally, we assume that the target of the reaching task is provided three seconds after starting the simulation (*t*_*T*_ = 3*s*). This allows us to inspect the behavior of the system at movement onset and to test the model stability in the absence of action triggers.

**Fig 7:**
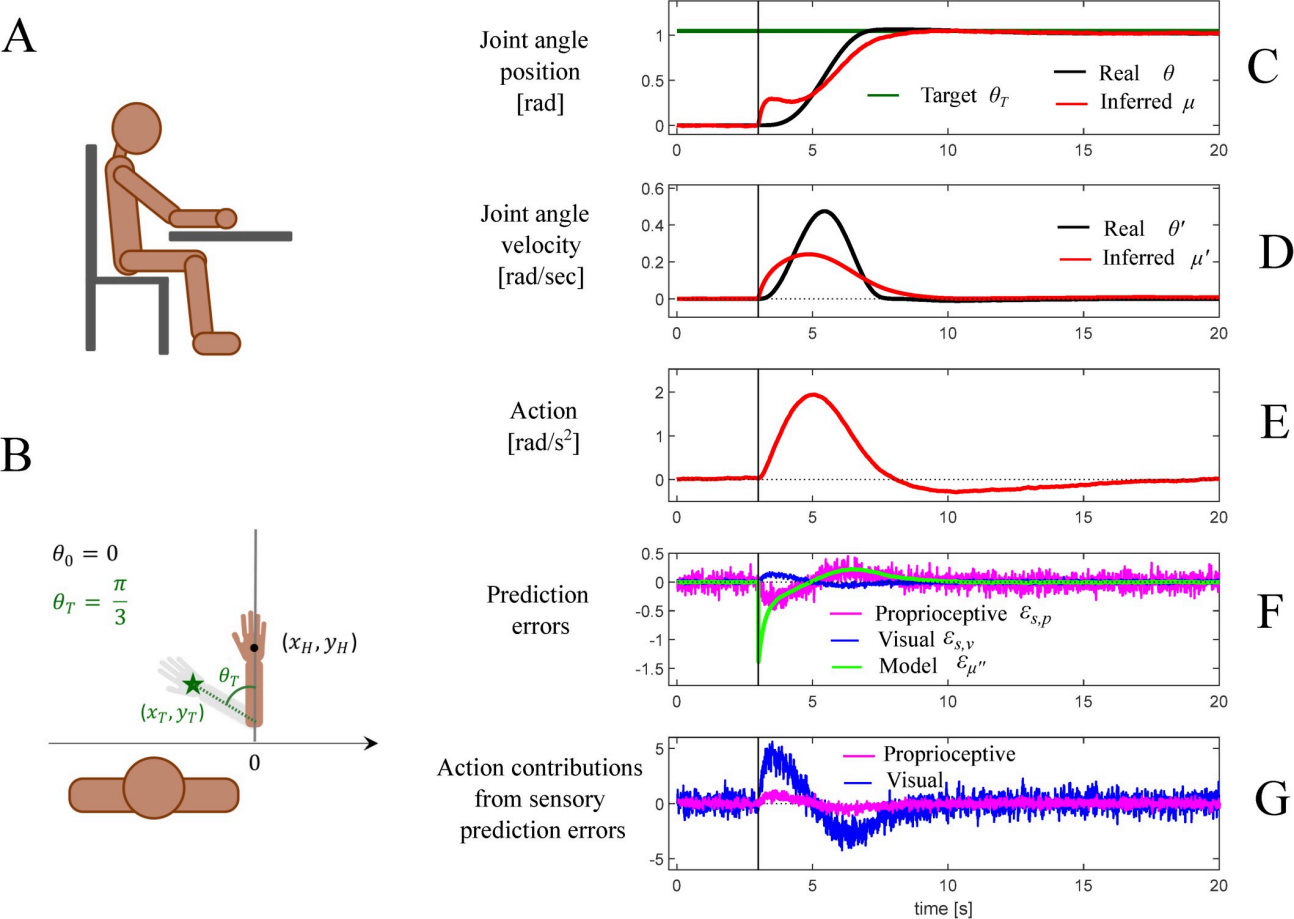
First simulation: reaching a fixed target in space. (A) Schematic representation of the agent having direct vision of his own arm, which initially rests on a table surface and could only move by rotating the elbow around the vertical axis. (B) Task specification. The agent has to reach a target location (green star) by rotating the arm so to reach the configuration shown in grey, at *θ_T_*, corresponding to having the hand at the target location [*x_T_, y_T_*] *= g(θ_T_)*. (C-G) Dynamics of the model variables during the task. The vertical bars mark the time at which the target location is disclosed. (C) Joint angle of the real (black) and inferred (red) arm configurations, expressed in radians; the green line represents the arm configuration *θ_T_* for which the hand is on the target. (D) Real (black) and inferred (red) velocity of the elbow joint angle velocity. (E) Action, represented in our model as an angular acceleration. (F) The three prediction errors considered in the model: model dynamics error (green) and the two sensory errors, proprioceptive (magenta) and visual (blue). Please note that in this plot, the prediction error units correspond to different dimensions and cannot be directly compared. (G) Contributions of proprioceptive (magenta) and visual (blue) errors in determining the action, more specifically the two vector components in the r.h.s. of Eq G4 in Fig 6. See the main text for explanation.

The simulation results are illustrated in Fig 7. Fig 7A shows a schematic of the simulated agent whereas Fig 7B shows the initial position of the hand and the target position (the green star) defining the task. Fig 7C–7G illustrate the temporal evolution of variables of the generative process and the generative model. Fig 7C and 7D show the temporal evolution of the two hidden states considered in this simulation—joint angle position and velocity, respectively—and highlight the differences between the “real” system state variables of the generative process (i.e., *θ* and *θ*′) and the corresponding state variables of the generative model (i.e., *μ*_θ_ and *μ*_θ′_). The next panels show the temporal unfolding of the generated action (Fig 7E); of the three kinds of prediction errors (the model prediction error and the two sensory prediction errors: visual and proprioceptive) (Fig 7F); and of the relative contributions of the two sensory prediction errors (visual versus proprioceptive) to the generated action (Fig 7G). The latter correspond to the two terms on the r.h.s of Eq G.4 in Fig 6. Please note that for illustrative purposes, we parameterized the system to simulate a slow reaching movement that lasts about 6 seconds.

In the first 3 seconds of the simulation, before the target is set, the agent keeps its posture. In this phase, prediction errors are on average null, beside fluctuations associated with sensory noise. Later on, when the target is set, the associated arm configuration (shown by the grey arm in Fig 7B) becomes the desired state, acting as an attractor in the internal model of the system dynamics. The internal estimate of the system’s state “drifts” towards the predicted dynamics, causing a sudden increase of the model prediction error (note that by definition *ε_μ′_* is always zero, so we do not report it in the following). Sensory prediction errors grow accordingly (Fig 7F), and eventually drive the action to reach the target.

Please note that the above pattern of results does not critically depend on the fact that the agent selects actions to minimize both visual and proprioceptive errors. A control simulation (S1 Fig) indicates that the same pattern of results can be obtained by assuming that the agent selects actions to minimize only proprioceptive errors, as assumed in previous active inference models of movement control [30,46]. Furthermore, similar results can be obtained by assuming that the agent has a biased representation of the sensory noises; for example, by assigning a higher precision to vision compared to proprioception ($\Sigma_{{\tilde{s}}_{p}}=0.3$ and $\Sigma_{{\tilde{s}}_{v}}=0.01$) or vice versa ($\Sigma_{{\tilde{s}}_{p}}=0.05$ and $\Sigma_{{\tilde{s}}_{v}}=0.03$).

### 3.2. Second simulation: multisensory conflict in the rubber hand illusion

**Fig 8:**
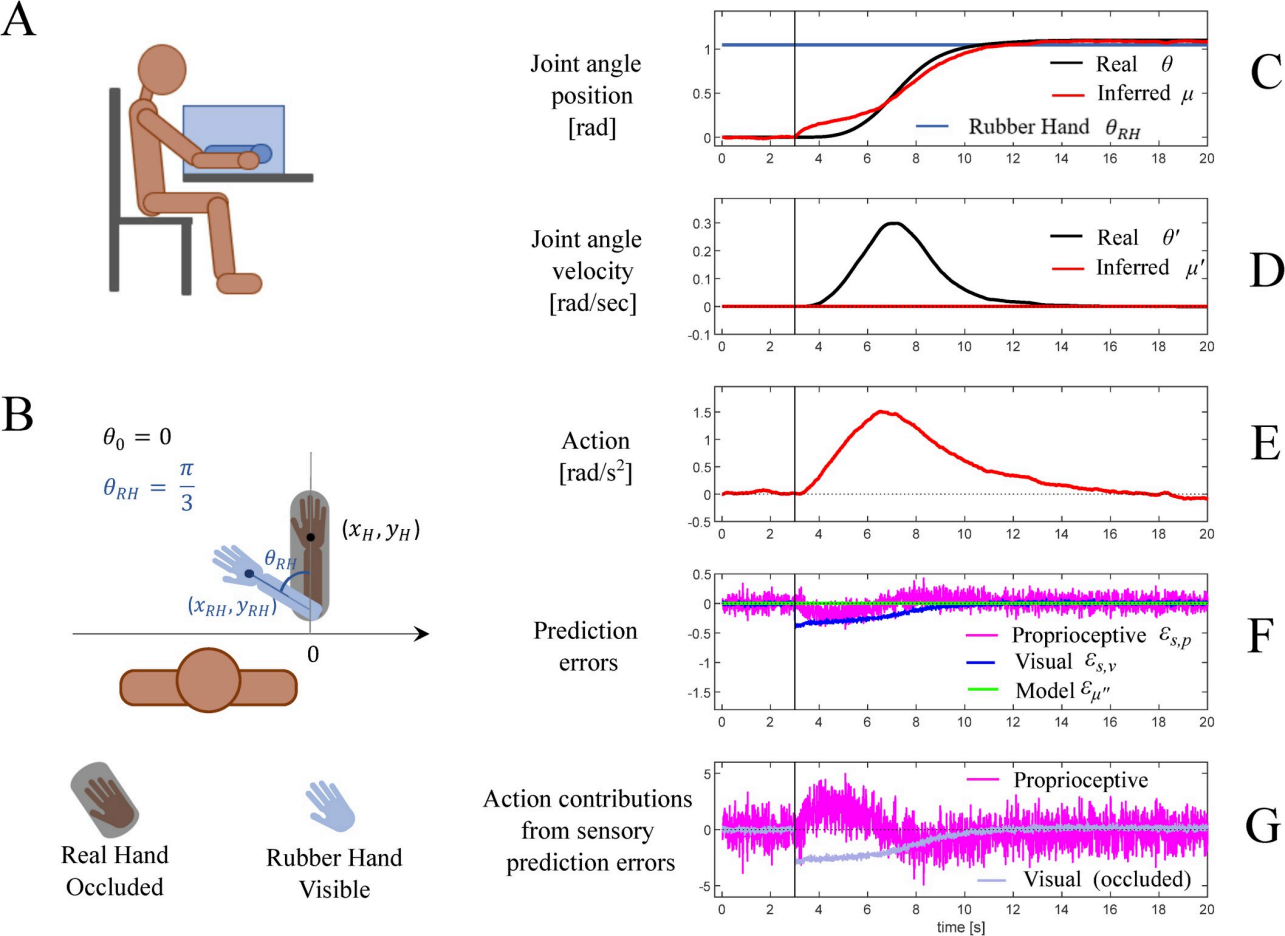
Second simulation: multisensory conflict in the rubber hand illusion (RHI). Schematic representation of the agent seeing a virtual hand through a virtual reality stereoscopic headset. (B) Task specification. The agent undergoes a body ownership illusion over a virtual arm shown with a rotation *θ_T_* with respect the real arm initial position. (C-G) Dynamics of the model variables during the task. The vertical bars mark the time of the illusion onset. (C) Joint angle of the real (black) and inferred (red) arm configurations, expressed in radians; the blue line represents the virtual arm configuration *θ_T_*. (D) Real (black) and inferred (red) velocity of the elbow joint angle velocity. (E) Action, represented in our model as an angular acceleration. (F) The three prediction errors considered in the model: the model dynamics error (green) and the two sensory errors, proprioceptive (magenta) and visual (blue). Please note that in this plot, the prediction error units correspond to different dimensions. (G) Contributions of proprioceptive (magenta) and visual (blue) prediction errors in determining the action, more specifically the two addends in the r.h.s. of Eq G4 in Fig 6. The gray line represents the potential visual contribution that is set off by assuming that *k_A,v_* = 0. See the main text for explanation.

In the second simulation, the active inference agent experiences the Rubber Hand Illusion (RHI) [11]. In the RHI, a static rubber hand misplaced with respect to the real hidden hand is processed as if being part of the self-body, thus the agent is exposed to a multisensory (visuo-proprioceptive) conflict. As for other Bayesian accounts of the RHI, we assume that the agent automatically attributes the sensory stimuli available (e.g. visual from the rubber hand and somatosensory from the real hand) to the same causal origin–the own body–and hence incorporates the rubber arm into his body representation. This assumption is reflected in the choice of the agent’s generative model that implicitly assumes that the visual input of the rubber hand originates from the physical hand. So, in the forward mapping of the generative model (Eq E.3) we assume that [*x*_*V*_, *y*_*V*_] = [*x*_*RH*_, *y*_*RH*_], where the latter vector represents the Cartesian coordinates of the rubber hand (see Fig 8B). However, differently from other Bayesian approaches to the RHI, active inference assumes that the agent can potentially resolve his multisensory conflict by moving the arm. To appreciate this possibility, we assume that the agent’s real hand is free to move.

We assume that the illusion is triggered instantaneously, after 3 seconds from the beginning of the simulation, and that the rubber hand is placed in the configuration corresponding to a joint angle of *π*/3 (for consistency with the first simulation). In this simulation we assume that action is driven exclusively by proprioceptive prediction errors and hence we set *k*_*A*,*v*_ = 0. This is because the agent has no control on the rubber hand and is therefore not able to change the visual input by moving. Finally, we assume that in this simulation, the agent has a lower visual precision of the hand location ($\Sigma_{{\tilde{s}}_{v}}=0.07$), reflecting the fact that it trusts the inputs from the rubber hand less than the inputs from the real hand. The low visual precision is also important to guarantee that the effects of the illusion arise gradually, despite for simplicity we assumed its onset to be instantaneous; see the Discussion for more details.

The simulation results are shown in Fig 8C–8G, with the same layout described for Fig 7. The model predicts that once the illusion is triggered, visual and proprioceptive streams provide incongruent information about hand position and hence generate sensory prediction errors that affect perceptual inference of the hand position, *μ_θ_*. In turn, to minimize the sensory prediction errors, the agent moves the real hand in the direction of the rubber hand. Please note that this occurs because in this simulation, moving the rubber hand in the direction of the real hand is impossible. If this were possible, e.g., keeping *k_A,v_* different from zero, we could have observed the opposite: a movement of the rubber hand in the direction of the real hand.

Importantly, as the agent does not have any explicit intention to move, the model dynamics errors are null. Given that the agent receives no model dynamics errors and has no sensory access to its velocity, it incorrectly infers that its velocity remains zero (Fig 8D), as if it executed no movement. This result provides a speculative explanation for the lack of awareness of movements that are executed in the absence of an explicit intention [51]. We will return to this point in the Discussion.

### 3.3. Third simulation: reaching under visuo-proprioceptive conflict

In the third simulation, the active inference agent has to reach a fixed target location with its right hand (as the first simulation) when it is exposed to a visuo-proprioceptive conflict (as in the second simulation). Different from the second simulation, the conflict originates from the fact that the agent is embodying the virtual arm of an avatar that moves in the same direction as the real hand, but faster: the movements of the real hand are mapped onto the virtual hand with a velocity gain of 1.3 along the same direction. This is done by extending the generative process with an additional dynamical variable, the virtual hand joint angle, θ_*VR*_, whose dynamics at the first order is set to ${\dot{\theta }}_{VR}=1.3\times \dot{\theta }$. This implies that any movement of the real arm corresponds to a faster movement of the virtual arm, producing a spatial misalignment between the two. Importantly, once the agent starts moving, a multisensory conflict is generated that cannot be resolved. This simulation therefore resembles classical studies of motor adaptation that modify sensorimotor mappings using (for example) fixed roto-translations [18].

We assume that at the beginning of the simulation, the virtual hand illusion is already established and the real and the fake hands are perfectly co-located: *θ_0_* = *θ_VR,0_* = 0. As in the first simulation, the target of the reaching task (*θ_T_* = π/3) is provided after three seconds (*t*_*T*_ = 3*s*). The other parameters are the same as the first simulation, too.

**Fig 9:**
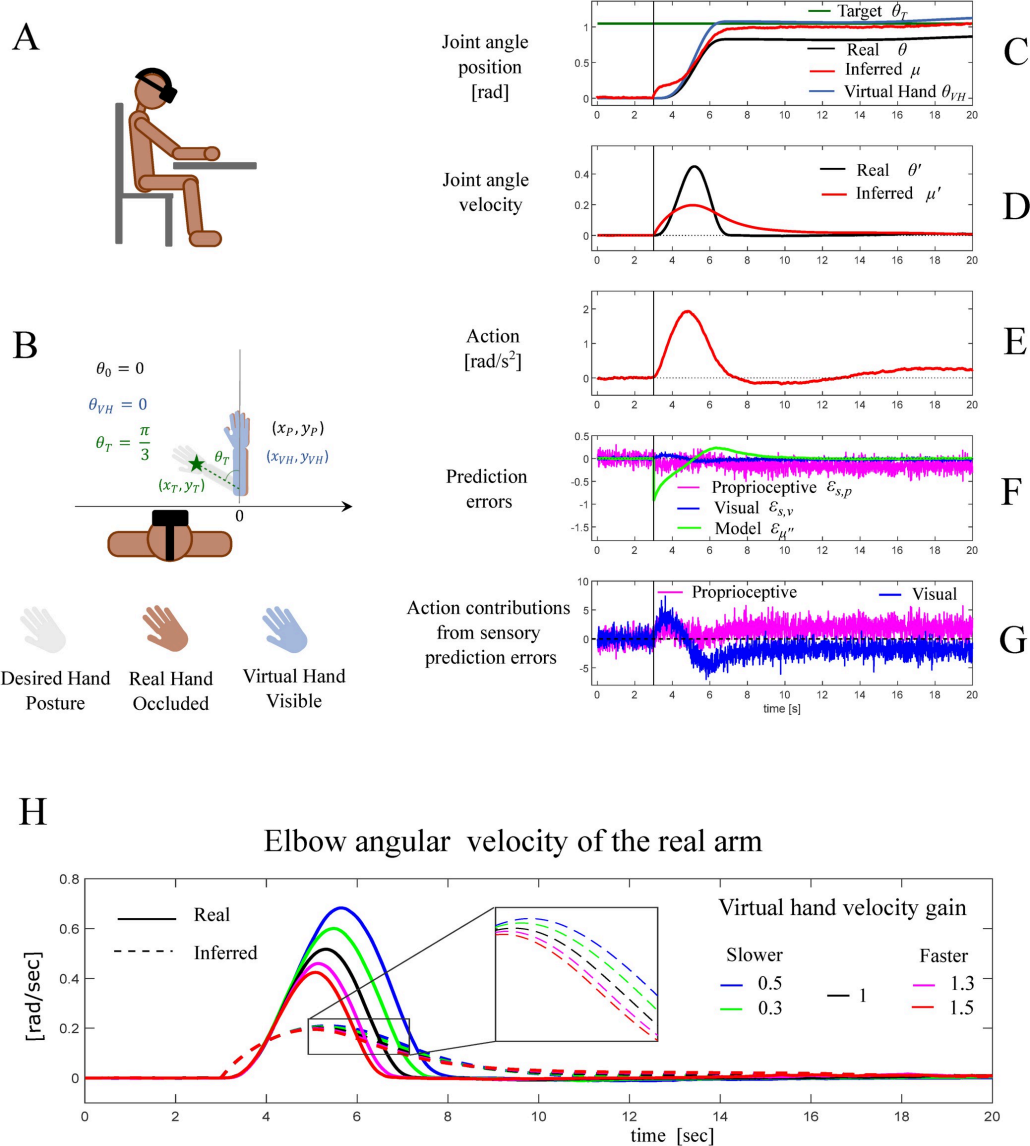
Third simulation: reaching under visuo-proprioceptive conflict that cannot be resolved by acting. (A) Schematic representation of the agent seeing a virtual hand through a virtual reality stereoscopic headset. (B) Task specification. The agent has to reach a target placed at [*x_T_, y_T_*] *= g(θ_T_*), while undergoing an illusory ownership over a virtual hand, which moves along the real hand but with a velocity gain of 1.3. (C-G) Dynamics of the model variables during the task. The vertical bars mark the time at which the target location is disclosed. (C) Joint angle of the real (solid black) and inferred (solid red) arm configurations, expressed in radians. The green line represents the desired arm configuration *θ_T_* for which the hand is on the target. (D) Real (black) and inferred (red) velocity of the elbow joint angle. (E) Action, represented in our model as an angular acceleration. (F) The three prediction errors considered in the model: model dynamics error (green) and the two sensory errors, proprioceptive (magenta) and visual (blue). Please note that in this plot, the prediction error units correspond to different dimensions. (G) Contributions of proprioceptive (magenta) and visual (blue) errors in determining the action, more specifically the two addends in the r.h.s. of Eq G4 in Fig 6. (H) Real (solid curves) and inferred (dashed curves) velocities of the elbow joint angle for different simulations that use different values for the velocity gain, which maps the real elbow joint angle velocity into the corresponding join angle velocity of the virtual arm. The black line, corresponding to a velocity gain 1, is the same as in the first simulation. The inset permits appreciating the differences between the inferred velocities. See the main text for a more detailed explanation.

The simulation results are detailed for the velocity gain of 1.3 in Fig 9C–9G, using the same layout as for Fig 7. In addition, we show the velocity profiles for different gains in Fig 9H. As in the first simulation, the target location acts as an attractor and influences the model dynamics, hence creating a model dynamics error. This, in turn, produces sensory prediction errors—that the agent minimizes by moving the arm towards the target location. However, in this simulation, once the agent starts moving, it experiences an additional multisensory conflict, because the virtual arm moves faster and becomes misaligned. Given the visuo-proprioceptive mismatch, the perceptual estimate of the hand location (red line in Fig 9C) shifts toward the virtual hand.

Because the virtual gain is larger than 1, the virtual hand moves faster and approaches the target ahead of the real hand. A steady configuration is then achieved when the inferred state, dominated by visual information from the virtual arm location, reaches the target. Note that, as vision is characterized by a higher sensory precision compared to proprioception, the inferred state is closer to the virtual hand and the real hand stops moving before reaching the target. This steady configuration, characterized by a null model prediction error, is sustained despite the persistent visuo-proprioceptive conflict. This is because the contributions of the visual and proprioceptive prediction errors to action counteract each other (Fig 9G), causing the overall action to be suppressed. In other words, since the real and the virtual hands lay on opposite sides with respect to the inferred hand location, there is no way of simultaneously reducing both visual and proprioceptive prediction errors. Moving the real hand towards the inferred state would reduce proprioceptive prediction error but also increase visual prediction error, because the virtual hand would be moved away from the inferred location. Conversely, moving the real hand away from the inferred location would move the fake hand towards it and hence reduce visual prediction error, but also increase proprioceptive prediction error. This result highlights the importance of including the contribution of visual prediction errors to action (see Eq G.3-G.4 in Fig 6). A control simulation (S2 Fig) illustrates that if the contribution of visual prediction errors to action is removed, the visuo-proprioceptive conflict cannot be resolved and then the real hand would continue to follow the virtual hand beyond the target, failing to comply with the assigned task.

Fig 9H shows the real (solid curves) and inferred (dashed curves) velocity profiles from different simulations, in which we used different values of the velocity gains that map the real joint angle velocity to the velocity of the virtual arm. The comparison between the magenta lines (corresponding to this third simulation, with gain 1.3) and the black lines (corresponding the first simulation, with no visuo-proprioceptive conflict and gain 1) is characterized by a clear shift that reflects the earlier damping in the join angle velocity discussed above. A similar, but more pronounced trend is observed when the velocity gain of the virtual arm is increased to 1.5 (red curve). When instead the virtual arm is set to move slower (green and blue curves), the real arm reaches the target before the virtual hand and–given that the virtual hand still lags behind–keeps moving beyond it, reaching its velocity peak later than in the other conditions and overshooting the target (Fig 9H). Interestingly, while the agent’s motor behaviour (solid curves) is clearly different in the five different visuomotor conditions of Fig 9H, the dynamics of the corresponding internal estimates of the arm velocity (dashed curves) shows minimal differences. This result suggests a possible reason why people are generally not aware of the small compensatory movements that they execute in conditions of subtle manipulations of the visuo-motor mapping [22].

In sum, this simulation illustrates that during visually guided actions with multisensory conflicts that cannot be resolved by acting, the intentional component largely dominates, obscuring the contribution of conflict-resolution mechanisms.

## 4. Discussion

Movement control is usually guided by an intentional imperative to reach external goals, as in the case of a person reaching an object with the hand. However, recent human-computer interaction and virtual reality studies revealed subtler aspects of motor behaviour that are not motivated by the achievement of external goals [13–15,17]. These include small motor adjustments that are driven by the need to reduce multisensory conflict between (for example) the real position of the hand and the position of an avatar’s hand, hence permitting to maintain a unitary body representation in the face of multisensory conflicts. Movements that obey the latter conflict-resolution imperative are usually ignored in mainstream models of motor control and difficult to reconcile with the necessity to achieve external goals [2,3].

Here, we presented a model grounded in the theory of active inference that provides a unitary perspective on the two main imperatives of motor control: the achievement of external goals and the resolution of multisensory conflicts. We presented three simulations that illustrated the capability of the active inference model to addresses the two imperatives and the similarities and differences between tasks that include external goals, multisensory conflict, or both. Below we highlight four key results of our simulations.

First, our simulations show that the same active inference model accounts for the achievement of external goals (first simulation), the resolution of multisensory conflicts (second simulation) and both imperatives simultaneously (third simulations). At difference with other approaches, such as optimal motor control [2] that only focuses on intentional aspects of movement, the framework of active inference considers multisensory conflict resolution as part and parcel of movement control. This means that it is not necessary to define ad-hoc functions to account for motor adjustments that occur in conditions of multisensory conflict. Rather, it is possible to derive them from first-principle accounts, such as (in active inference) the minimization of variational free energy [29,30,37,49,52–54]. Previous works showed that it is possible to simulate various aspects of movement control under multisensory conflict, including pathological behaviour [31,55]. Here, we show that we can reproduce both intentional and conflict-resolution imperatives of motor control in the same model, across three simulations. Crucially, this does not require changing the generative model or its dynamics, but only to adapt the model parameters to reflect task demands.

Second, our simulations highlight a crucial difference between movements that are triggered by intentional and conflict resolution imperatives. To understand this point, it is worth reminding that active inference considers two kinds of prediction errors: model dynamics errors that stem from a discrepancy between the inferred and the expected model states; and sensory errors (visual and/or proprioceptive) that stem from a discrepancy between predicted and sensed observations. While action is only driven by sensory errors, model dynamics errors can trigger it indirectly, by driving the perceptual inference away from the current sensory input and thus producing sensory prediction errors. Crucially, the distinction between actions triggered with or without model dynamics errors becomes key to understand the differences between intentional and conflict-resolution movements. Intentional actions arise because of changes in model dynamics that produce model dynamics errors. During reaching, for example, setting a target for movement changes the dynamics of the model and drives the expected state towards the target. In turn, this generates sensory prediction errors that drive intentional movements in the direction of the target—and cancel out not just sensory errors, but also the model dynamics errors that generated the sensory errors in the first place. Instead, conflict-resolution movements arise in the absence of model dynamics errors. Bodily illusions (or aberrant self-perception) can directly trigger sensory prediction errors in the agent’s forward model that maps the system state into (visual and proprioceptive) sensory channels. For example, in the RHI, visual inputs from the rubber hand are processed as if originating from the body. The virtual hand location is then merged with the position sensed by proprioception. This leads to an inferred state at an intermediate location between the two, which is used in the internal forward model ***g***_***μ***_ to compute sensory predictions, thus producing sensory prediction errors in both the visual and proprioceptive domains. In standard RHI experiments, when the participant cannot move the hand, this would result in a so-called proprioceptive drift, which marks an incorrect estimation of the hand location [11,23]. In our simulations, when the agent is able to minimize prediction errors by moving, the sensory prediction error produces an action towards the rubber hand, in agreement with what observed experimentally under similar conditions [13]. This is in line with the results from [14], derived from an active inference model specifically tailored to simulate the RHI.

Third, while we emphasized the distinction between intentional and conflict-resolution imperatives of motor control, our simulations show that both imperatives might be simultaneously in play and their relative impact on movement may vary, depending on task demands. Specifically, when multisensory conflicts can be resolved by acting (second simulation), the necessity to resolve the conflicts triggers movement even in the absence of external goals, as observed during bodily illusions [13–15,17]. Rather, in conditions traditionally addressed by sensory adaptation studies [18,24], when the action is goal-directed and the multisensory conflict cannot be resolved by acting, the intentional component dominates, obscuring conflict resolution. The fact that the contribution of conflict-resolution to movement control is often obscured by the intentional imperative might explain why the former has been received scant attention so far. However, besides bodily illusions, the conflict-resolution imperative might potentially help understand other apparently puzzling phenomena in motor control, such as the so-called "ideomotor" movements [56,57] that are triggered by the observation of others’ actions and the aberrant action awareness in some psychopathologies (see below).

Fourth, our simulations lead to the speculative proposal that if action is only driven by conflict-resolution, the agent does not update its estimate (and does not become aware) of its action velocity, see Fig 8D. This is because in our model the agent cannot directly sense its velocity but only infer it, on the basis of the model dynamics error, which is however absent when action is only driven by conflict-resolution. Hence, when the agent is compensating for visuo-motor conflicts in the absence of external goals, such as during the RHI, it incorrectly infers that is not moving. To the extent that awareness is associated with this (velocity) inference, this finding provides a novel (albeit speculative) explanation for the lack of motor awareness associated to compensatory movements during embodiment and virtual reality studies [58,59]. Please note that this result critically depends on the assumption that the agent does not receive any reliable observation about its velocity but can only infer it based on model dynamics error or through changes in muscle length, via type Ia fibers, whose responses are however inhibited for slow movements [60,61]. If the agent were able to observe its velocity, as assumed in other works [55], it would be able to update its velocity estimate even in absence of model dynamics errors. It is also worth noting that the lack of motor awareness when action is driven by conflict resolution is different from the lack of awareness of compensatory movements executed during goal-directed actions. As reported by a large body of literature, people are generally unaware of the small compensatory movements that they make while reaching a target that is shifted subtly [22]. Furthermore, when the target position or the visual feedback from the hand is shifted abruptly, people firstly make compensatory movements and become aware of these movements only afterwards [62,63]. In all these cases, given that the action is goal-directed, a shift of the target would produce a model dynamics error and hence in principle the agent should be able to infer that it is moving. However, it is possible that when model dynamics errors are introduced smoothly (as in [22]), they are not sufficient for motor awareness. Rather, larger and abrupt model dynamics errors, as those associated with target or arm perturbations during reaching actions [62,63], can raise awareness, but with a slower time course compared to the induced compensatory movements. This raises the possibility that in the latter case, motor awareness reflects the (retrospective) inference that one has moved, more than the (prospective) inference that one is moving or about to move. Still another case is the lack of motor awareness associated to psychopathological conditions, such as the alien hand syndrome [64], phantom limbs [65] and anosognosia, where patients experience the illusion that they can move their paralysed limb [66]. In these conditions, an incorrect inference that one is moving (or not moving) could arise from a model that does not properly monitor its model errors and hence relies too strongly on prior expectations, which in turn fail to be updated in the light of novel evidence and become excessively rigid [51,67]. This idea would align well with computational theories of various other psychopathological conditions, such as panic disorder [68], psychosis [69] and eating disorders [70,71], which focus on incorrect prioritization (and precision-weighting) of prior information and prediction errors during inference [72]. All these are speculative hypotheses that remain to be tested in future studies.

The current model has some limitations that could be addressed in future studies. The first limitation is that in our second and third simulations, we assume that the agent is automatically under the effect of a bodily illusion and incorporates the hand into the body representation. A more detailed simulation could include the modelling of the illusory ownership of the virtual hand, e.g., with Bayesian inference approaches implemented in previous studies [9,39,43]. This could be done by including an additional (hierarchically higher) layer of inference that adjudicates between two hypotheses, namely, that the visual and proprioceptive information come from a common cause versus different causes. The latter hypothesis would correspond to a lack of rubber hand illusion (or a lack of "embodiment" of a virtual avatar) that plausibly arises when the multisensory conflict is excessive; for example, when the position of the fake hand is implausible. This is similar to what happens in multisensory integration experiments, in which visual and auditory stimuli could be perceived as unified if their spatial or temporal disparity is not excessive, and not unified otherwise [73]. On the other hand, it is noteworthy that once illusory ownership is in place and the agent has inferred a common cause for its sensations, the spatiotemporal constraints for multisensory integration are relaxed, allowing for an increased degree of misalignment [74]. Using a hierarchical approach provides also the possibility to account for the fact that the illusory ownership of a fake hand or body can have different intensities (stronger or weaker) rather than being dichotomous (illusion or not illusion). The graded nature of bodily illusions can be modelled by considering the probability of the hypothesis that there is a common cause for all sensations, with a higher probability corresponding to a stronger illusion and a lower probability corresponding to a weaker illusion. In this case, the sensory prediction errors associated with the forward mapping ***g***_***μ***_ linking all sensory input, could be weighted according to the probability associated to the "common cause" hypothesis, as proposed in [14]. This method would allow us to model "weak" forms of embodiment illusion, in which compensatory movements and perceptual recalibration could occur even in the absence of explicit reports of body ownership. Finally, the hierarchical approach permits modelling a putative role of prior information about the common cause in modulating the intensity of the illusion.

The hierarchical approach–and especially the possibility to tune sensory prediction errors depending on the degree of illusory ownership–would help resolving a second limitation of the current model. In the second simulation, we assumed that the agent automatically tunes two parameters of the internal model. First, the agent cancels the contribution given by the visual prediction error to action, given that it does not have control over the rubber hand. Second, the agent decreases its visual precision. The low visual precision dampens the effect of vision in multisensory integration on the internal state estimate, which guarantees that the proprioceptive drift originating from the illusion arises gradually. A gradual arise of the proprioceptive drift is important in our setup, because if the agent infers an instantaneous "jump" of the hand location towards the fake hand, it would have to compensate for abrupt sensory prediction errors, which in turn could produce instabilities in motor control. An alternative (and perhaps more appealing) way to realize a gradual onset of the illusion without lowering visual precision would be using a hierarchical approach to modulate the degree of illusory ownership. This hierarchical approach would reduce the magnitude of visual prediction errors not because vision is assigned low precision, but because the agent does not fully trust the fact that the fake hand is part of its body. This idea remains to be tested in future studies.

The third limitation of the current model is that it does not fully explain in which conditions a multisensory conflict is resolved with perceptual recalibration, compensatory movements, or both. While most existing models of multisensory conflicts during bodily illusions emphasize proprioceptive recalibration [23,43,44], here we have shown that an active inference agent can also cancel out the conflict by making compensatory movements. If during a RHI (or similar experiment) the agent cannot move, the only available solution is perceptual recalibration. Rather, if the agent can move, there is an initial perceptual recalibration that is followed by a mandatory compensatory movement, which in turn abolishes the perceptual recalibration. However, in a more realistic scenario, perceptual recalibration and compensatory movements could be modulated by task demands and even coexist. This is the case, for example, if compensatory movements are only executed when sensory prediction errors are above a threshold (e.g., when they surpass the energetic costs for movements). More broadly, in real life conditions, the respective contributions of perceptual recalibration and compensatory movements to multisensory conflict resolution would depend on the model’s precision parameters, which could lead to subtle dynamics [59,75,76].

The fourth limitation is that in the simplified 1DoF model presented here, we could not simulate the simultaneous unfolding of motor behaviours driven by the intention and the conflict-resolution imperatives, as observed in the self-avatar follower effect [17]. This would require extending the model to include two or more degrees of freedom, in order to realize a null space in which movements of the real arm do not affect the state of the virtual limb. Similarly, future studies could investigate how the choice of specific model parameters impacts its dynamics. Finally, another open objective for future research future studies is assessing how well the active inference model proposed here account well for human behaviour in the three tasks considered here and how it compares to other models in motor control that consider control costs related to the minimization of uncertainty [20].

In sum, we advanced a computational model of movement control grounded in the framework of active inference that unifies its two imperatives–intentional and conflict-resolution–that were studied in isolation so far. The contribution of the latter imperative is obscured in real life conditions but it becomes apparent in studies where multisensory conflicts can be manipulated flexibly, as during virtual reality and body ownership illusions, as well as during psychopathological conditions. Our study therefore contributes to shed light on aspects of movement control whose goal is to maintain a self-consistent perception of the body, which in turn is a key precondition for the achievement of externally specified goals.

## Supporting information

S1 Appendix The appendix briefly summarizes the mathematical formulation of active inference used in this study. (PDF)

S1 Fig Reaching a fixed target in space. The plots show the results for a control simulation in which we replicate the first simulation (whose results are shown in Fig 7 of the main text) but setting k_p_ = 0.6 and k_v_ = 0, i.e. assuming that the agent selects actions to minimize only proprioceptive errors only. Panel from top to bottom show the same variable shown in Fig 7C–7G.
(TIF)

S2 Fig Reaching under visuo-proprioceptive conflict that cannot be resolved by acting. The plots show the results for a control simulation in which we replicate the third simulation (whose results are shown in Fig 9 of the main text) but setting k_p_ = 0.6 and k_v_ = 0, i.e. assuming that the agent selects actions to minimize only proprioceptive errors only. The figure shows the same variable shown in Fig 9C.
(TIF)
