# Supplementary material for: Active inference unifies intentional and conflict-resolution imperatives of motor control
Source: PLoS Comput Biol. 2022 Jun 17;18(6):e1010095. doi: 10.1371/journal.pcbi.1010095 (PMC9205531; doi:10.1371/journal.pcbi.1010095)
Supplement: S1 Appendix — (PDF) [file pcbi.1010095.s001.pdf]

Supplementary Materials

Active inference unifies intentional and conflict-resolution imperatives of motor control

Antonella Maselli[1], Pablo Lanillos[2], Giovanni Pezzulo[1*]

1. Institute of Cognitive Sciences and Technology, National Research Council (CNR), Rome, Italy.

2. Donders Institute for Brain, Cognition and Behaviour, Artificial Intelligence Department, Radboud University, Nijmegen, The Netherlands.

*giovanni.pezzulo@istc.cnr.it

Appendix

In this section, we briefly summarize the mathematical formulation of the free energy principle and the ensuing active inference formulation of perception and action. Please see [1–3] for additional details.

The free energy principle has been formulated to provide a unifying theory of perception and action in biological organisms and their associated neuronal underpinnings. At its root the free energy principle embraces the Helmholtzian perspective, according to which perception, action and other cognitive processes are the result of a probabilistic inference about the hidden causes of sensory signals. In the classical Bayesian Inference formulation, this process is equivalent to computing the posterior probability of the hidden causes, x, given the sensory signals available, s, as give by the Bayes rule:

$$p(x|s) = \frac{p(s|x)p(x)}{p(s)}$$

Here $p(s|x)$ represents the likelihood of the sensory signals for a given configuration of the environment (and thus of the hidden causes x) an $p(x)$ represents the prior expectation on the state of the environment that the agent holds before receiving sensory information. The normalization factor at the denominator, $p(s)$, represents the marginal likelihood (obtained marginalizing the likelihood over all possible states), which is in most applications is intractable.

Variational free energy [4] has been introduced as an approximation of Bayesian Inference problems that require computing the posterior probability of the hidden causes, $p(x|s)$. The variational approximation is based on the idea that an inferring agent holds an approximate representation of the posterior probability $p(x|s)$, known as the recognition density, $q(x)$, which can be computed by minimizing the Kullback-Leibler (KL) divergence between the two distributions:

$$\boldsymbol{D_{KL}}\,(q(x)||\,p(x|s)) \;\coloneqq\; \int \frac{q(x)}{p(x|s)}dx$$

Note that the latter equivalence corresponds to the formal definition of KL divergence, which quantifies the difference between two probability distributions. Although the KL divergence as defined above cannot be derived exactly, it can be rewritten as:

$$\boldsymbol{D_{KL}}\,(q(x)||\,p(x|s)) \;=\; \int q(x)\log\frac{q(x)}{p(x,s)}dx + \int q(x)\log\frac{p(x,s)}{p(x|s)}\,dx \quad \coloneqq\; \mathcal{F} \;+\; ln\,p(s)$$

Where the last equivalence results from the formal definition of the variation free energy $\mathcal{F}$ (first terms in the central and left sides), and to the fact that the logarithm in the second integral can be rewritten as $\log p(s)$ and kept out of the integration (the remaining integral of the recognition density over all possible states is 1 by definition of probability distribution). From the last equation, it appears clear that minimizing free energy with respect to the recognition density is equivalent to minimize the KL divergence between the posterior probability and $q(x)$ (as the second term in the r.h.s of the last equation is a constant), and therefore to optimize the inference problem. The advantage of introducing variational free energy is that under a set of plausible assumptions, it is possible to derive an analytical formulation of $\mathcal{F}$, which could be then minimized through gradient descent methods.

By definition, the variational free energy can be rewritten as:

$$\mathcal{F} = -\int q(x) \log p(x,s) \ \ dx \ + \ \int q(x) \ \log q(x) \ \ dx \ .$$

In principle, $q(x)$ could take any form, but in order to keep the mathematical problem tractable, *q(x)* is assumed to be a multivariate Gaussian specified by its sufficient statistics (i.e., by the expected means and standard deviations $[\ \mu_i, \Sigma_i\ ]$, with $i \in [1, N]$ spanning the N variables that describe the system state). With the additional assumptions that the joint probability is a smooth function of x and that its logarithm can be approximated with a quadratic function near the mode, the variational free energy can be expressed in terms of the Laplace encoded energy $L(s,\mu) \ \coloneqq \ -\log p(s,\mu)$:

$$\mathcal{F} \approx L(s,\mu) + \sum_i \frac{1}{2} (\log \Sigma_\mathrm{i} \ + n_i ln 2\pi)$$

The two assumptions above are also known as the Laplace approximation, and the full derivation of the resulting simplified expression of $\mathcal{F}$ have been demonstrated in previous works [5–7].

If one further assumes that Σ_i are constant, minimizing $\mathcal{F}$ with respect to the recognition density $q(x)$ is equivalent to minimizing $\mathcal{F}$, and therefore the Laplace encoded energy $L(s,\mu)$, with respect to μ_i. The second term was thus, in the mathematical model description in Fig 5 referred to the constant *C*. The variational free energy can be then expressed by working out a treatable (derivable) expression of the Laplace-encoded energy − and of the joint probability $p(s,\mu)$ − where the inferred state of the environment is approximated with the sufficient statistics of the recognition density. For the problem treated in this paper and the related model implementation, this is done throughout the equations in Fig 5.

Once the approximated formulations for the variational free energy have been derived, the problem of perceptual inference can be approached computationally by estimating the value of μ that minimizes $\mathcal{F}$, and therefore maximizes the joint probability of sensory data and associated internal state estimate (by definition of Laplace encoded energy), which is akin to Bayesian formulations of perception. In addition, the active inference formulation postulates that agents can minimize the free energy (and therefore maximize $p(s,\mu)$) by performing actions that affect the sensory state and can thus drive the free energy along a gradient descent. The detailed implementation of the action-perception loop adopted for our model is described in Figure 5. Crucially, the perceptual inference driven by the integrated effect of the internal system dynamics and the free energy minimization has a direct impact on the actions that are generated internally as a strategy to further minimize free energy. The actions in turn, eventually affect the dynamics of the environment.

As discussed in the main text, computing actions as part of the internal model dynamics requires an additional caveat. In fact, the internal model of the system, and therefore the expression derived for the variational free energy, do not have an explicit representation of the action. For this, active inference formulations assume that agent holds an implicit knowledge about how its actions affect the sensory state. This means that in order to select actions that minimize the free energy, the impact of action on $p(s,\mu)$ should be computed indirectly, by considering how actions affect the sensory states and how the sensory states affect the joint probability $p(s,\mu)$. An example of this, for the specific case of the model implemented in this study, is provided in equations G.3 and G.4 of Fig 6.

References

1. Friston K, Daunizeau J, Kilner J, Kiebel SJ. Action and behavior: a free-energy formulation. Biol Cybern. 2010;102: 227–260. doi:10.1007/s00422-010-0364-z

2. Parr T, Pezzulo G, Friston KJ. Active Inference: The Free Energy Principle in Mind, Brain, and Behavior. Cambridge, MA, USA: MIT Press; 2022.

3. Oliver G, Lanillos P, Cheng G. An empirical study of active inference on a humanoid robot. IEEE Trans Cogn Dev Syst. 2021; 1–1. doi:10.1109/TCDS.2021.3049907

4. Beal MJ. Variational algorithms for approximate Bayesian inference. University of London; 2003. Available: http://www.cse.buffalo.edu/faculty/mbeal/papers/beal03.pdf

5. Friston K, Mattout J, Trujillo-Barreto N, Ashburner J, Penny W. Variational free energy and the Laplace approximation. NeuroImage. 2007;34: 220–234. doi:10.1016/j.neuroimage.2006.08.035

6. Opper M, Archambeau C. The Variational Gaussian Approximation Revisited. Neural Comput. 2009;21: 786–792. doi:10.1162/neco.2008.08-07-592

7. Buckley CL, Kim CS, McGregor S, Seth AK. The free energy principle for action and perception: A mathematical review. J Math Psychol. 2017;81: 55–79. doi:10.1016/j.jmp.2017.09.004
