# Supplementary figures and images for: Active inference unifies intentional and conflict-resolution imperatives of motor control
Source: PLoS Comput Biol. 2022 Jun 17;18(6):e1010095. doi: 10.1371/journal.pcbi.1010095 (PMC9205531; doi:10.1371/journal.pcbi.1010095)

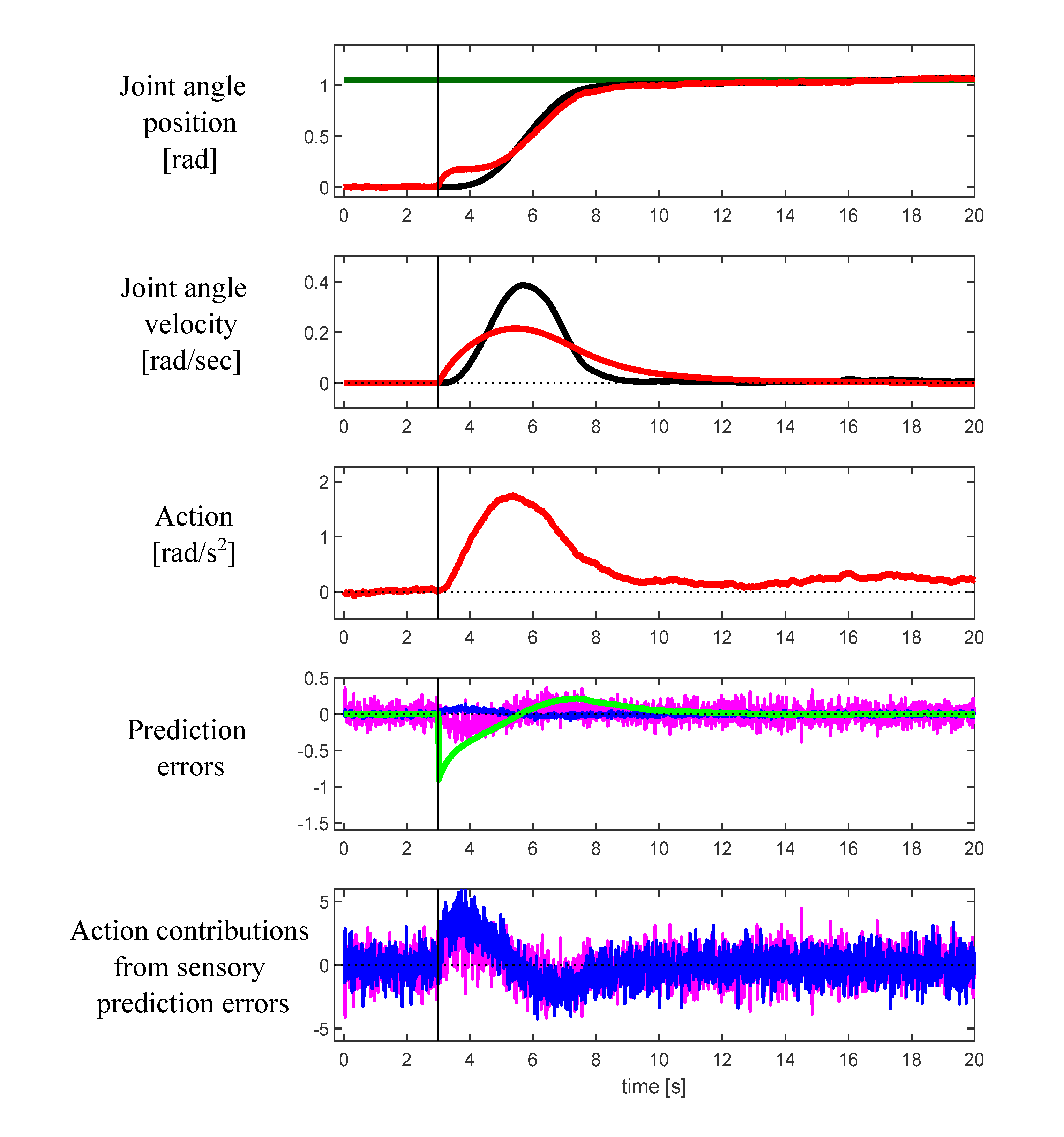

Supplement: S1 Fig — The plots show the results for a control simulation in which we replicate the first simulation (whose results are shown in Fig 7 of the main text) but setting kp = 0.6 and kv = 0, i.e. assuming that the agent selects actions to minimize only proprioceptive errors only. Panel from top to bottom show the same variable shown in Fig 7C–7G. (TIF) [file pcbi.1010095.s002.tif]

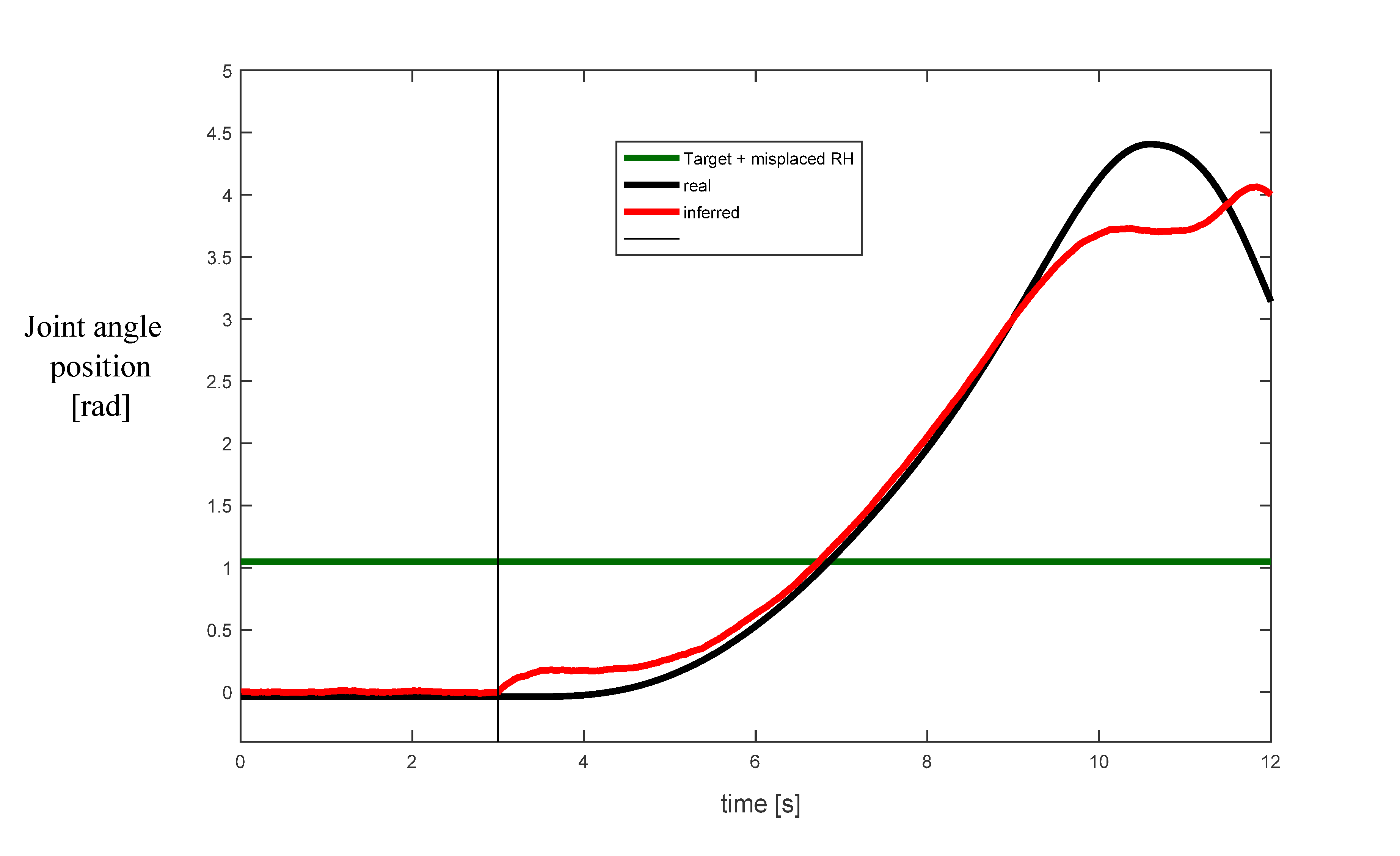

Supplement: S2 Fig — The plots show the results for a control simulation in which we replicate the third simulation (whose results are shown in Fig 9 of the main text) but setting kp = 0.6 and kv = 0, i.e. assuming that the agent selects actions to minimize only proprioceptive errors only. The figure shows the same variable shown in Fig 9C. (TIF) [file pcbi.1010095.s003.tif]
